# Functional near-infrared spectroscopy in pediatric clinical research: Different pathophysiologies and promising clinical applications

**DOI:** 10.1117/1.NPh.10.2.023517

**Published:** 2023-03-03

**Authors:** Anne Gallagher, Fabrice Wallois, Hellmuth Obrig

**Affiliations:** a CHU Sainte-Justine University Hospital, Université de Montréal, LIONLab, Cerebrum, Department of Psychology, Montréal, Quebec, Canada; b Université de Picardie Jules Verne, Inserm U1105, GRAMFC, Amiens, France; c University Hospital and Faculty of Medicine Leipzig/Max Planck Institute for Human Cognitive and Brain Sciences, Department of Neurology, Clinic for Cognitive Neurology, Leipzig, Germany

**Keywords:** children, adolescents, pediatrics, epilepsy, language disorders, attention-deficit/hyperactivity disorder, functional near-infrared spectroscopy

## Abstract

Over its 30 years of existence, functional near-infrared spectroscopy (fNIRS) has matured into a highly versatile tool to study brain function in infants and young children. Its advantages, amongst others, include its ease of application and portability, the option to combine it with electrophysiology, and its relatively good tolerance to movement. As shown by the impressive body of fNIRS literature in the field of cognitive developmental neuroscience, the method’s strengths become even more relevant for (very) young individuals who suffer from neurological, behavioral, and/or cognitive impairment. Although a number of studies have been conducted with a clinical perspective, fNIRS cannot yet be considered as a truly clinical tool. The first step has been taken in this direction by studies exploring options in populations with well-defined clinical profiles. To foster further progress, here, we review several of these clinical approaches to identify the challenges and perspectives of fNIRS in the field of developmental disorders. We first outline the contributions of fNIRS in selected areas of pediatric clinical research: epilepsy, communicative and language disorders, and attention-deficit/hyperactivity disorder. We provide a scoping review as a framework to allow the highlighting of specific and general challenges of using fNIRS in pediatric research. We also discuss potential solutions and perspectives on the broader use of fNIRS in the clinical setting. This may be of use to future research, targeting clinical applications of fNIRS in children and adolescents.

## 1 Introduction

The potential of near-infrared spectroscopy (NIRS) to noninvasively assess oxygenation changes in the cerebral cortex was initially considered to be of primarily clinical relevance. Undoubtedly a “pulse oximeter” to monitor the brain’s well-being would be a valuable tool in intensive neuro-care situations that include the monitoring of sick infants and children. Early work showed some success.^1^^–^^3^ However, the establishment of a true clinical application in adults is still debated,^4^^,^^5^ whereas, notably, the largest potential thus far is in critical care settings for infants and neonates.^6^^,^^7^ In parallel to the clinical perspective, there has been growing interest in NIRS in the functional imaging community. The advent of new techniques, especially functional magnetic resonance imaging (fMRI) and positron emission tomography (PET), which rely on the focal hemodynamic response to localize hubs of large-scale functional networks, has spurred the use of NIRS as an alternative neuroimaging tool. This methodology has been used in nearly every field of cognitive neuroscience and the ever-increasing number of publications based on “functional” near-infrared spectroscopy (fNIRS) has established fNIRS as an easy-to-use, comparatively inexpensive, noninvasive alternative option to investigate the functional architecture of the cerebral cortex. This is particularly true for “special populations,” which may not be easily examined in the highly constraining fMRI/PET environments. The impressive body of work on fNIRS in clinical cognitive developmental neuroscience in infants and children illustrates that, in addition to global measurements of the brain’s well-being, focal activation patterns could be used to understand the pathophysiology of a number of disorders in this age group. Ideally, indicators of imminent deterioration or precursors of later developmental challenges could warrant intervention to minimize persistent impairment.

Indeed, fNIRS has been used to study multiple clinical populations with various conditions and neurodevelopmental disorders, mostly to better understand the neuropathophysiology of the disease. fNIRS studies have provided important findings to the research and clinical communities. Regardless of the pathology studied, it is essential to consider cerebral dysfunction/function in both their neuronal and vascular dimensions. This is readily provided by fNIRS, which offers relatively good temporal and spatial resolution and can be easily combined at the bedside with other modalities, such as electroencephalography (EEG). However, to date, few studies have led to advances in diagnosis or intervention strategies for pediatric conditions and neurodevelopmental disorders. This can be explained by various methodological limitations and challenges of fNIRS.

In this perspective paper, we outline the contributions of fNIRS in several selected topics of pediatric clinical research. We do not provide a full systematic review of the topics, nor do we intend to cover the full spectrum of conditions in infants, children, and adolescents. Instead, in each section, we first provide a scoping review of the state of fNIRS research and clinical applications. We then highlight certain specific challenges associated with the clinical condition and briefly discuss perspectives for fNIRS in the field.

We start with fNIRS research in epilepsy (Sec. 2), which is linked to the hypersynchronization of neuronal populations expressed either as interictal spikes or seizures that affect nearby and distant neuronal and vascular networks. Epileptic syndromes in infants and children are partially shaped by the maturational trajectory of subcortical and cortical tissues and their connections. The first part focuses on the neurovascular unit. This expresses our view that (pathological) changes in the interaction between the neuronal and vascular compartments in the developing brain can be considered to be of supreme importance in understanding the pathology and intervening accordingly. In the subsequent two sections, we focus on two key cognitive capacities that start to develop very early in life. The common challenge is that the effect of pathologically altered development may only become behaviorally relevant later in life. This is why early multimodal assessment is of paramount relevance. We then discuss the fNIRS literature on language and communication disorders (Sec. 3). Due to the large number of papers on the effect of cochlear implants on hearing and the perception of spoken language, these are included here. Although language is not restricted to the auditory modality, early linguistic development is strongly driven by auditory input. This is addressed in the reviewed papers. In the following section, we review the fNIRS literature on attention-deficit/hyperactivity disorder (ADHD, Sec. 4), which is thought to be caused by the dysfunction of integrative neural networks. In addition to the intrinsic differences of the underlying pathophysiologies, the choice of these three topics was also motivated by the fact that they target different age groups. The neurovascular assessment of epilepsy can be considered to be particularly important in prematurely born neonates and infants. Although starting at birth, the developmental impairment of language—the most important tool for communication and social participation—typically becomes apparent around the age of 2 or 3 years, whereas ADHD is rarely diagnosed before the child enters school or even later. In the last sections of this article, we briefly discuss the overall challenges and limitations, as well as potential perspectives, of using fNIRS in pediatric research and clinical settings.

### 1.1 Methodology and Searching Strategy in PubMed

**Fig. 1:**
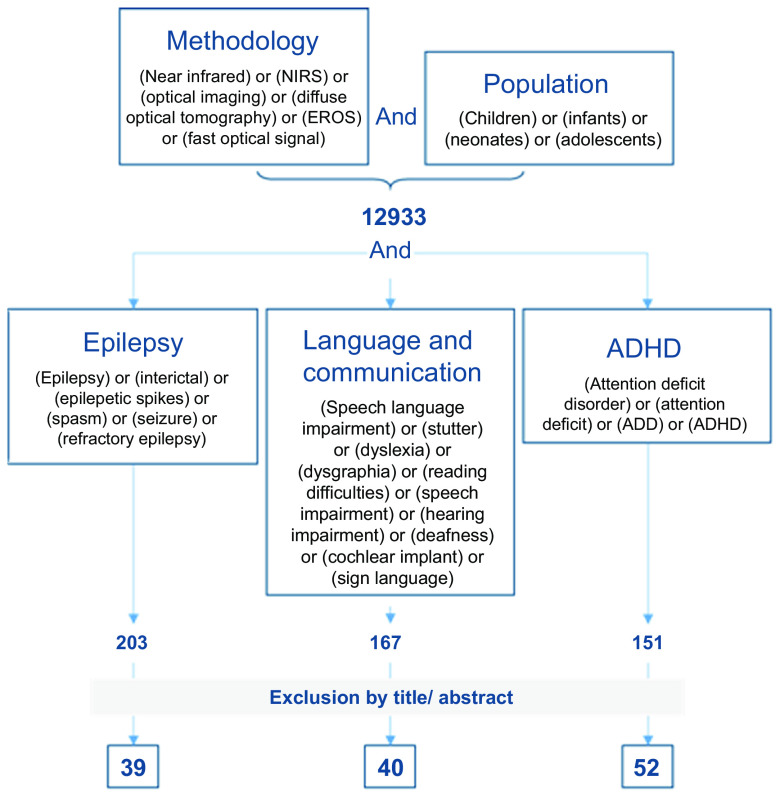
Flowchart illustrating the selection of articles in the scoping review process. Search terms for the methodology and population (n=12,933 hits) were combined with search terms for the three pediatric conditions. The abstracts/titles were then scanned to exclude false and/or redundant hits. The remaining studies formed the basis for review, Secs. 2–4. They are listed in Tables S1–S3 in the Supplementary Materials.

We established a search strategy and include summary tables (see Tables S1–S3 in the Supplementary Materials) to provide a global idea of the state of fNIRS research on the three selected topics. To cover different approaches of NIRS, we used the following search terms for the methodology: [(near infrared) or (NIRS) or (optical imaging) or (diffuse optical tomography) or (EROS) or (fast optical signal)]. this was linked to the search terms for the targeted pediatric population [(neonates) or (infants) or (children) or (adolescents)]. these terms were linked to the search terms for each clinical condition (as specified in fig. 1 and in each section): (methodology) and (population) and (clinical condition). The search strategy and selection process for the three sections are illustrated in Fig. 1.

## 2 fNIRS and Epilepsy in Pediatric Populations

Epilepsy is one of the most frequently occurring childhood neurological diseases. It presents as the activation of a pathological neuronal network caused by an imbalance between excitatory and inhibitory influences, essentially resulting in pathological endogenous hypersynchronization. Such hypersynchronization constitutes the dysfunctional basis for the emergence of seizures and interictal epileptic spikes. The extensive endogenous temporal and spatial recruitment of neural networks induces a well-characterized hemodynamic response revealed by functional MRI^8^ and a metabolic response demonstrated using clinical single-photon emission computed tomography (SPECT; ictal and interictal) and PET (interictal). Nevertheless, these hemodynamic and metabolic imaging techniques are difficult to implement in pediatric populations and lack temporal resolution. NIRS, coupled with electroencephalography (EEG)/electrocorticography (ECoG), offers a feasible approach for children, with good temporal resolution of vascular dynamics. This allows monitoring of the modifications of the energy environment that surround both the interictal spikes and the various types of seizures. It provides a better understanding of the pathophysiological mechanisms and may improve the management of epileptic children, particularly in situations of refractory epilepsy that require surgery. In this section, we will successively approach the work carried out to date using EEG/ECoG-NIRS to study interictal spikes and seizures and to identify the language eloquent zones in the presurgical assessment of drug-resistant epilepsy in children.

### 2.1 What has been Studied?

The PubMed review search yielded 175 hits. The search terms for this section were “epilepsy” or “interictal” or “epileptic spikes” or “spasm” or “seizure” or “refractory epilepsy.” After screening the abstracts to remove false and or redundant hits, 39 articles remained. Half were published before 2013. All articles are listed in Table S1 in the Supplementary Material. They can be grouped into three classes: (1) NIRS and interictal manifestations (epileptic spikes and epileptic discharges), (2) NIRS and epileptic seizures, and (3) NIRS and the lateralization/localization of language function in potential surgical candidates with refractory epilepsy. In the next sections, we will present an overview of the main results extracted from these papers.

#### 2.1.1 Interictal spikes

The studies can be separated into two groups. One group of studies investigated the pathophysiological mechanisms related to the emergence of epileptic spikes. Both fast optical signals (e.g., fast optical signals or “FOS”^9^) and oxygenation-based fNIRS (i.e., assessing changes in Hb-chromophores) were used, FOS for its excellent temporal resolution, making it possible to target the neuronal components, and fNIRS for its relatively good temporal resolution, allowing analysis of the hemodynamic changes surrounding the epileptic spikes. The fNIRS studies highlighted an increase in HbO and a decrease in HbR within the 10 s following the epileptic spikes,^10^^,^^11^ irrespective of whether the epileptic spikes were temporo-mesial (n=3) or neocortical (n=16). Hemodynamic changes preceding the epileptic spikes by a few seconds^11^ were also observed, confirming in children what had been described in animals^12^^–^^14^ and humans using fMRI.^8^ Hemodynamic changes were also recorded at distant sites from the epileptogenic zone, as observed with fMRI, confirming the involvement of a neuronal network rather than a single hub causing the hemodynamic changes associated with epileptic spikes, regardless of the type of epilepsy or species.^11^ In some cases, the observed hemodynamic diffusion was accompanied by changes in neuronal synchronization distant from the epileptic focus, resulting in dysfunction secondary to the occurrence of the epileptic spikes (e.g., cognitive dysfunction in benign epilepsy with centrotemporal spikes, BECTS). Similarly, changes in scattering related to changes in membrane configuration were observed when measuring FOS at epileptic spikes. This work suggests that changes in the extracellular space precede the emergence of the spikes by 500 ms.^15^ Such modifications of the extracellular space support the idea of a modification of the availability of neurotransmitters around epileptic spikes. Overall, because of their excellent/good temporal resolution, FOS and fNIRS have demonstrated that the epileptic spike is not an isolated element but rather an element in an energetic context and a particular extracellular environment.

The other group of publications comprises methodological studies targeting better detection of epileptic spikes in the NIRS signal. Initially, hemodynamic changes related to epileptic spikes were analyzed either by simple averaging or by using a GLM transfer function, as used in fMRI studies. The main problem is that the fNIRS response is highly variable due to the epileptic spikes, in particular, when the hemodynamic changes occurring prior to the emergence of the spike are considered.^13^ Considering the nonlinearity of the hemodynamic response function (HRF) in children and adults with refractory epilepsy by applying a deconvolution method specific for each patient (Volterra methods with gamma functions) improves the sensitivity of the detection of hemodynamic changes related to the epileptic spikes.^10^^,^^16^^,^^17^

#### 2.1.2 Seizures

Seizures are classified according to the International League Against Epilepsy (ILAE).

##### Neonatal seizures

Neonatal seizures, like any other type of seizure, are paroxysmal, repetitive, and stereotypical events. They are usually clinically subtle, inconspicuous, and difficult to distinguish from the normal behavior of the interictal periods or physiological phenomena. In full-term neonates suffering from postanoxic encephalopathy, NIRS results consistently differentiate states in which the EEG tracing is flat, devoid of background activity^18^ or not.^19^ In both cases, a decrease in the tissue oxygenation index (TOI) was associated with an increase in HbO and an initial increase in HbR, followed by a decrease. This hemodynamic response has been shown to originate from the brain, as it was not found in the signal of short-distance channels.^18^ This suggests an initial increase in the metabolic rate of oxygen (${\mathrm{CMRO}}_{2}$), with an increase in $O_{2}$ consumption during the seizure that is greater than that during physiological activation. Thus, in such an anoxic situation in full-term neonates, the increase in local blood flow may not fully compensate for the increase in oxygen consumption.^18^^,^^19^ In neonatal intensive care units (NICUs), this may aid the evaluation of the adaptation to seizures and contribute to clinical decision algorithms to identify risk factors of such seizure discharges.

##### Focal seizures

Focal seizures are those with a focal onset beginning on one side of the brain. We identified six studies that included nine child participants across them, pooled together with adults.^20^^–^^25^ Watanabe et al.^20^ only reported an increase in cerebral blood volume (CBV), in agreement with the hyperperfusion observed with SPECT, whereas Nguyen et al.^23^ and Gallagher et al.^22^ reported typical neurovascular coupling, with an increase in HbO in parallel to a decrease in HbR in the initial phase of the seizures. Nguyen et al.^23^ computed a lateralization index of the hemodynamic response. This may be useful to better localize the epileptogenic zone in refractory epilepsy. Although the lateralization index adequately lateralizes the epileptic focus at the group level, it failed in one out of the three children who participated in the study. Nevertheless, in the past decade, fNIRS has been increasingly used simultaneously with EEG and in conjunction with other techniques (e.g., fMRI, SPECT, and PET) to localize the epileptogenic zone in a presurgical context, and now contributes to clinical surgical decisions for some patients. Another study^24^ reported patients (n=4) with complex partial seizures in which two patients presented an increase in HbO and HbR for the duration of the seizure of up to 4 min, whereas no changes were recorded for the two remaining patients.^25^ This suggests progressive recruitment of the vascular system as the neuronal network becomes engaged in the seizure process. This was confirmed by the informative study of Sato et al.,^26^ who combined ECoG and simultaneous cortical fNIRS for a child suffering from a brain tumor. The child had seizures originating in the supplemental motor area. An increase in cerebral blood flow was reported over the seizure onset zone, starting about 2 s after ECoG seizure onset, around 6 s before clinical seizure onset, and with rapid hemodynamic propagation to adjacent areas in agreement with the ECoG propagation. During surgery, such an approach could provide useful information about the seizure onset zone and seizure propagation at the cortical level.^26^

##### Spasms

An epileptic spasm consists of brief (1 to 3 s) events of arm, leg, and head flexion (arms and legs pull into the body) or extension. Spasms typically occur in clusters, with events every 5 to 10 s over a 5-to-10-min period. Cortical and subcortical structures are involved in the mechanisms that trigger epileptic spasms. Spasms in West syndrome patients have been shown to elicit an increase in CBV associated with an increase in HbO, HbR, and HbT.^24^ Three different patterns of regional increases in CBV were identified in a series of spasms (a) repetitive transient Hb increases synchronized with the spasms, (b) a gradual focal increase associated with fluctuations during a cluster of spasms that were synchronized with each spasm, and (c) a transient periodic increase that also contained elements resembling a gradual increase. Evaluating six patients with epileptic spasms of different etiologies,^27^ a two-phase stereotypical hemodynamic response was described. The use of short channels to differentiate the impact of the hemodynamic skin response suggests a truly cerebro-cortico-subcortical origin of the described pattern. The first phase consisted of an increase in CBV, with an increase in HbO and HbR, followed by the second phase, characterized by typical neurovascular coupling (increase in HbO + decrease in HbR) that increases progressively with the emitter-detector distance. In one case, the coupling was inverted. In this case, the origin of the seizures was a metabolic disorder. In yet another case, the neurovascular coupling response was absent, as expected in anencephaly. Overall, the data suggest that the first increase in CBV is related to subcortical structures involved in the initiation of spasms.^27^^,^^28^ These results are consistent with the hypothesis that a number of specific complex processes in subcortical cortical loops are involved in every single event of a series of spasms.^27^ Due to the relatively good temporal resolution of NIRS, this fits well with the existing hypothesis that subcortical primary lesions can cause epileptic spasms in certain West syndrome patients.

##### Absence seizures

Absence seizures are characterized by a transient brief loss of consciousness. They are accompanied by EEGs by a discharge of spikes and waves at 3 Hz with a rapid onset and offset. In a first report^24^ that included two patients, one showed no hemodynamic changes during the seizure, whereas the other showed a decrease in HbO and an increase in HbR. In a study including 29 children with absence seizures^29^ and another by Nourhashemi et al.,^30^ generalized spike wave discharges (GSWD) were found to be associated with a change in oxygenation starting 20 s before seizure onset. This was characterized by an initial decrease in cortical oxygenation followed by a more classical neurovascular response consisting of an increase in HbO starting 10 s prior to and peaking 5 s after seizure onset, with subsequent deoxygenation. Moreover, HbO and cerebral blood flow (CBF), monitored by diffuse correlation spectroscopy, negatively correlated with the EEG DC-shift in the frontal region and positively correlated in posterior regions. This suggests that EEG DC-shifts observed in absence seizures are associated with changes in HbO and CBF.

##### Seizure detection

In addition to the characterization of the hemodynamic response to the hyperactivity of seizures, NIRS could be useful for better detecting seizures in a clinical environment during patient monitoring. This idea was investigated by Sirpal et al.,^31^ who showed that multimodal neuroimaging, such as EEG-fNIRS, in epileptic patients could enhance seizure detection in a group of 40 patients, including five teenagers.

#### 2.1.3 Characterization of language eloquent areas and lateralization prior to epilepsy surgery

Four papers addressed language lateralization during noninvasive presurgical evaluation and three investigated whether “language eloquent areas” can be detected by intraoperative hemodynamic (fNIRS) and electrophysiological approaches. During the testing of verbal fluency, receptive language, and/or performance in syllable repetition tasks in six children, fNIRS data were recorded over Broca’s area using 128 channels matched intracarotid amobarbital test (IAT) results for all individuals. This suggests that fNIRS has the potential to be a viable noninvasive alternative to IAT and fMRI in the determination of speech lateralization in the presurgical evaluation of children and adults.^22^^,^^32^^,^^33^ A case report^34^ compared fNIRS-EEG and fMRI results for a child with left temporal epilepsy and demonstrated the importance of coregistration of EEG with fNIRS to adequately localize language in children with epilepsy. This procedure is now being routinely used in clinical practice for patients with refractory epilepsy in pediatric hospitals in Montreal, Canada. Interestingly, the research group also developed a new approach for language mapping in children using resting state fNIRS connectivity (fcNIRS).^35^ Comparison of the fcNIRS approach to task-based fNIRS showed a good concordance between the two approaches for language lateralization. fcNIRS may be especially well-suited for the assessment of language lateralization in not fully participative populations, such as young children or patients with severe language or cognitive impairment. In epileptic patients, ECoG applied simultaneously with fNIRS has also been used to provide insights into the cortico-cortical networks underlying language function. Direct cortical stimulation of Broca’s area in three children evoked a well-localized typical hemodynamic response in Wernicke’s area via cortico-cortical connectivity. Such an approach developed in the operating room could contribute to neurosurgical decisions when typical language eloquent areas must be considered for surgery.^36^^,^^37^

### 2.2 What are the Challenges?

Investigating epilepsy using simultaneous EEG/ECoG and fNIRS includes the challenges of multimodal and multidimensional data acquisition and analysis. From a technical point of view, it is necessary to further develop tools that allow for the simultaneous recording of the two modalities with complementary spatial (fNIRS) and temporal (EEG) resolution without electrical crosstalk between the two modalities (notably electrical artifacts on EEG arising from NIRS optodes). This requires the development of medical devices and caps that support both electrodes and optodes adapted to age and recording conditions, in intensive care for example, with the capacity of dealing with high-density configurations in both modalities. Although multidimensional (neuronal and vascular) data are challenging, combining EEG/ECoG and fNIRS makes it possible to address the dysfunction/function of several compartments: neuronal (EEG), vascular (fNIRS), and intercellular (FOS). fNIRS investigations in the context of epilepsy should always include a simultaneous EEG or ECoG recording and video monitoring to identify epileptogenic activity and for off-line artifact rejection (e.g., body movements, sucking, etc.). The challenge consists of the integration and simultaneous analysis of multidimensional information, which can be useful for a better understanding of the dynamics of the different sectors in the emergence of interictal spikes and seizures at the group level. The challenge for clinical applications is to also supply information at the individual level to improve individual care. This may necessitate high temporal and spatial resolution for simultaneous EEG/ECoG recordings. Another challenge is the assessment of neuronal networks that include nonsuperficial hubs. EEG/ECoG and fNIRS suffer from poor spatial resolution in deep structures, in particular, in children. Therefore, because epilepsy is a network pathology, the lack of information about dysfunction in deep structures is an important limitation. In addition, the lack of observed neurovascular coupling in interictal epileptic spikes (IES) or epileptic seizures may be related to the poor sensitivity of fNIRS or an insufficiently high density of optodes to extract the hemodynamic information. Nevertheless, EEG and NIRS provide information from two different compartments, neuronal and vascular. The relationships between these compartments have been largely debated (see Ref. 38 in pediatric epilepsy). Changes in neuronal synchronization (hypersynchronization in epilepsy) do not always imply an increase in metabolism, whereas an increase in the firing rate of a large population of neurons is likely to modify the energy needs in the region of epileptic discharge. Finally, the change in HbO within 5 s reflects an average of what occurs at the neuronal level within at least 1 s, knowing that the duration of hypersynchronization of the IES is ∼500  ms, which is flanked by decreases in synchronization.^39^ The resulting hemodynamic effect may be negligible.

### 2.3 What are Potential Perspectives?

The main potential of fNIRS coupled with EEG lies in its ability to monitor the neuronal, vascular, and intercellular compartments. Technically, this requires medical devices that combine the two modalities at the sensor scale (electroptodes^®^ TM;^40^). This also implies developing diffuse optical tomography strategies^37^ that allow spatial resolution at the surface of the cortex comparable to that of 3T fMRI when using diffuse optical tomography with a high density of optodes.^37^^,^^41^ Epilepsy is certainly a pathology linked to the endogenous hypersynchronization of populations of neurons that are propelled into a pathological spiral. However, this occurs in a particular hemodynamic and metabolic environment that should be monitored for both interictal epileptic spikes and seizures. Moreover, while EEG and ECoG only monitor the synchronous activation of well orientated pyramidal neurons, fNIRS makes it possible to evaluate the vascular dynamics resulting from neuronal activation of not only pyramidal cells or interneurons but also of glial cells. This does not only pertain to the activation of hypersynchronous neurons but also unsynchronized neuronal activation, provided that this is coupled to increased oxygen supply.^40^ The concordance of information between the different compartments may converge at the individual level. For example, neuronal desynchronization and vascular deactivation in BECTS in the frontal regions could be used to evaluate the impact of epileptic spikes on transient cognitive deficits. By carefully monitoring such neurophysiological signatures, the impact of therapeutic management could be observed at a distance from the epileptogenic zone. Moreover, FOS makes it possible to monitor the dynamics of the extracellular space and therefore the availability of neurotransmitters at different levels of pathological neuronal networks. Improving our knowledge about such fine-tuning between different compartments could pave the way for novel therapeutic management and the development of new treatments. At the individual level, particularly in the management of drug-resistant epilepsy in children, this would allow for a better definition of the epileptogenic zone to be resected, as well as of the eloquent zones that need to be protected. These measurements can be easily performed repeatedly at the patient’s bedside through the scalp and in the operating room directly from the cortex. Thus, despite the relatively small number of publications in the field of childhood epilepsy, fNIRS coupled with EEG is still an interesting approach that could lead to clinical developments, including high-density strategies and real-time signal processing tools.

## 3 Developmental Disorders Affecting Language and Communication

Cognitive development during infancy, childhood, and adolescence strongly depends on social interaction and communication. This entails that developmental disorders that affect the most powerful communication tool, language, interfere with typical development. Linguistic and more general cognitive development strongly interact with each other, and disturbances can occur at different developmental stages; although basic aspects of linguistic development are already present at birth,^42^ full linguistic competence is reached only in late adolescence.^43^ Moreover, linguistic development comprises both “automatic” processes (e.g., tuning into the phonetic inventory of a given language) and the acquisition of skills in the framework of formal instruction (e.g., literacy [Of note, the research on literacy is biased towards industrialized, monetarily richer societies. Other social conventions of communication are acquired by instruction, but these have not yet been the focus of fNIRS-based research.]). More generally, human language development is believed to emerge from the intimate interaction between nature and nurture.^44^ Human brains appear to soak up linguistic input from or even prior to birth. In typically developing infants, the extraction of regularities in the auditory input is one of the foundations for the development of linguistic competence in close interplay with more general cognitive development. In impaired language development, this implies that any condition that affects the perception and/or processing of linguistic input from a very early age will potentially lead to severe deficits in later language competence.

The options to use fNIRS in this field are diverse. For uncompromised language development, an impressive spectrum of linguistic phenomena has been targeted (for reviews^45^^–^^47^). In this field of developmental neurolinguistics, fNIRS has the advantage of being noninvasive, low-constraint, and silent compared to fMRI and can be easily combined with EEG. The latter two advantages should be highlighted in the context of research on language development because (i) the auditory presentation of language stimuli is necessary for all preliterate participants and (ii) the long established and broad use of event-related potentials in developmental neurolinguistics has yielded neurophysiological markers that correlate with developmental milestones.^48^ Similar to EEG, the methodology is comparatively well-tolerated by infants and children and can be applied in a setting close to the natural “habitat” of even very young participants. This is one of the reasons why the methodology has established its place in developmental neurophysiology.

It stands to reason that the application of fNIRS in developmental disorders should profit from the cornucopia of developmental neurolinguistic findings published in the past two decades. However, a number of challenges and limitations must be respected when using this methodology in a clinical context. Of note, in the field of neurolinguistics, FOS to study changes in optical properties related to changes in the membrane configuration of activated neurons has been used in research on adults only.^49^ For infants and children, most studies have used fNIRS to target the much slower hemodynamic response. Whether or not the hemodynamic response pattern is different in infants relative to adults has been debated^50^^,^^51^ and has been recently reviewed, including issues of stimulus design.^52^

### 3.1 What has been Studied?

The scoping review search yielded 167 hits. The search terms used in this section were: ((speech language impairment) or (stutter) or (dyslexia) or (dysgraphia) or (reading difficulties) or (speech impairment) or (hearing impairment) or (deafness) or (cochlear implant) or (sign language)). After screening the abstracts to remove false and or redundant hits, 40 articles remained, which are listed in Table S2 in the Supplementary Material.

Since auditory input is provided from birth, and even intrauterine auditory signals may shape precursors of language development,^53^ severely impaired hearing will strongly affect later language development. It is thus notable that the largest number of pediatric fNIRS studies have focused on the effect of cochlear implants (CIs) on hearing and the perception of spoken language. The first study^54^ demonstrated the high detectability of a bilateral fNIRS-response to segments of auditorily presented stories. Although largely a feasibility-study, detection of an fNIRS response in 82% of normal-hearing children and 100% of hearing adults is a good starting point to investigate alterations of the fNIRS response in CI-users. Further advantages of the study’s design for potential clinical use were an undemanding sparse-channel approach and a state-of-the-art analysis technique allowing for robust detection of cortical activation. Other studies have confirmed the feasibility of this approach and have addressed cross-modal plasticity as a factor in success of CI users to adapt to the device. As no physical or artifact-interference occurs with fNIRS in CI-users, its suitability for the study of this population has been highlighted in five review papers. However, the only study truly addressing potential clinical relevance is that of Wang et al.^55^ Using a longitudinal design with two sessions within 6 months after unilateral CI-implantation, the study showed no difference between the implant side in terms of the fNIRS response to linguistic input, but an advantage for left-CI-implantation for the perception of nonlinguistic material. This is consistent with established models that show that speech comprehension depends on bilateral perisylvian cortices.^56^ The authors concluded that the generally recommended right-CI-implantation may not be justified if only unilateral CI-implantation is possible due to limited resources or medical reasons. Such a direct clinical recommendation is remarkable in the field, as most studies have described activation patterns without a direct consequence on clinical decisions.

Concerning language perception and comprehension, a number of studies have confirmed the potential of fNIRS to detect a response in infants with developmental deficits and in prematurely born children. An impressive example of the use of fNIRS in very early language development is provided by a study showing that even in children prematurely born three months before term, a linguistic (phonemes /ba/ versus /ga/) and voice contrast (male versus female) are differentiated by the premature infant’s brain.^57^ In an interesting follow-up study, the group was able to show that the EEG response to the contrast could be elicited in preterm infants who suffered from an intraventricular hemorrhage, whereas a hemodynamic response was not detected, showing deviations from typical neurovascular coupling in this pathology.^58^ This and other studies underline the high versatility of fNIRS in research on early language development. However, more clinically oriented applications targeting later language development are scarce. This is partially due to the wide range of tasks that have been used, targeting the many facets of emerging linguistic competence. It may be necessary, however, to establish “standard” stimulation procedures to assess how the individual child’s brain processes language. This may allow for the use of neurophysiological markers, including fNIRS measurements, in clinical research on the diagnosis, prognosis, and/or therapy-related changes of (imminent) speech-language impairment. Established and simple linguistic contrasts should be used^57^ and future research may profit from findings using event-related potentials (ERPs) to describe milestones in developmental neurolinguistics.^48^ A number of authors have highlighted this limitation in the literature reviewed here. Most prominently, a study assessing the validity of typical baseline conditions for auditory language tasks used in fMRI and EEG (time-reversed speech, TR, or noise correlated to the speech signal, SCN) showed that they may not be suited to fNIRS experiments.^59^ The authors tested 25 children of ∼9 years of age and compared fNIRS responses to spoken sentences to responses to TR or nerve conduction velocity. Although a response over auditory areas was successfully measured in most children, no lateralization or a statistically significant difference between speech and either of the nonspeech baselines was found. Although other researchers successfully used these baselines (e.g., for TR^60^) the study highlights potential limitations when stimulation paradigms from other imaging modalities are transferred to fNIRS research.

For speech “production,” several studies have addressed alterations in the cortical activation pattern in individuals who stutter. During development, 5% to 8% of preschool children develop such speech dysfluencies, but stuttering persists in only ∼10% of these children. Therefore, a major challenge in this field is to predict whether a child who stutters will or will not spontaneously recover. While most fNIRS studies have described the altered cerebral activation patterns in children who stutter relative to their nonaffected peers, Hosseini et al.^61^ showed that such a challenge can also be addressed by studying neurophysiological signals supplied by fNIRS. Using data from a previous study,^62^ the group developed a classifier that detected children who stutter in 87% of cases when based on fNIRS data. The classifier was trained on the data of 46 children, including children who stutter, children who showed no speech disfluencies, and a group of children who had recovered from stuttering. Eighteen channels covering the bilateral superior temporal and inferior frontal areas were used for all children. Of interest in the context of the current paper, application of the classifier to children who recovered from stuttering classified them as “no-stutter.” Although this may simply reflect a state difference at the time of the fNIRS measurement, it is a first promising step toward applying such a classifier to children who stutter and correlating it with a later outcome (recovery versus persistence of stuttering).

A small number of studies have addressed “dyslexia.” Although one of the most common developmental disturbances, the published papers using fNIRS are methodologically weak (see Table S2 in the Supplementary Material for details of the studies). For example, one study selectively measured oxygenation changes over a left prefrontal area to claim that the differential oxygenation response in dyslexic children relative to a control group indicates deficient working memory.^63^ Working memory depends on a large network and changes in the fNIRS response cannot be a surrogate marker for the assessment of such a basic cognitive function. Similarly, the claim that the difference in oxygenation response “causes” the working memory deficit is not justified. Studies targeting network functions, such as working memory, need to measure more than one of the “cortical hubs” and should be very careful to not confuse correlational and causal relationships when interpreting the data.

In summary, fNIRS has been successfully used to demonstrate its applicability in various developmental disorders/disturbances of language and communication disorders. Recent studies mostly targeting feasibility and/or questions concerning the underlying pathophysiology have attempted to explore the prognostic options of fNIRS measurements in children with CIs and those who stutter. In the next section, we will highlight the respective challenges for such applications.

### 3.2 What are the Challenges?

Targeting diagnostic or prognostic functional imaging markers of the developing brain is like walking on shifting sand. This is true for the assessment of all cognitive functions and clinical populations, including children with language and communication disorders and those with attentional disorders, as discussed in the following section on ADHD (Sec. 4). Due to the constitutive high plasticity in this age range, a specific contrast may only be indicative of later development within a very narrow age window. Similarly, the concept of “critical periods” during language and cognitive development assumes that infants may easily acquire specific skills in a specific age window and will have great difficulties to do so at later stages of development. Concerning language, an impressive, albeit debated, example is the “vocabulary spurt”^64^^,^^65^ referring to the exponential growth of the word knowledge of children starting at around 20 months of age. Although such “windows” may exist, it is also important to acknowledge the large interindividual variance. In summary, longitudinal assessment may be necessary. The ERP literature has demonstrated that electrophysiological “milestones” of language development can be defined.^48^

One of the challenges of using fNIRS to study language and communication is the movement artifacts induced when the child is performing an expressive language task. The choice of method for the identification and correction (or rejection) of movement artifacts is important and may differ between the type of artifact and populations. Careful signal inspection is, therefore, important and necessary.

A study also investigating speech perception highlighted the caveat concerning the use of fNIRS without adequate control for extracerebral signal contribution (see Ref. 66 for a demonstration of the stimulus-locked autonomic response in adults). In this study,^67^ the changes in heartbeat were extracted from recordings of a 44-channel fNIRS monitor of infants (3 to 12 months of age) with a high (n=40) versus low risk (n=48) of having autism spectrum disorder (ASD). The results showed group differences between baseline heart rates. More importantly, the heart rate response to stimulation (three-syllabic pseudowords) also differed between the two groups. This finding may be valuable for research on the physiological differences between high- versus low-ASD-risk children. However, it also highlights that systemic hemodynamic parameters can be stimulation-correlated and need to be corrected when functional cerebral activation is targeted.

### 3.3 What are the Potential Perspectives?

**Fig. 2:**
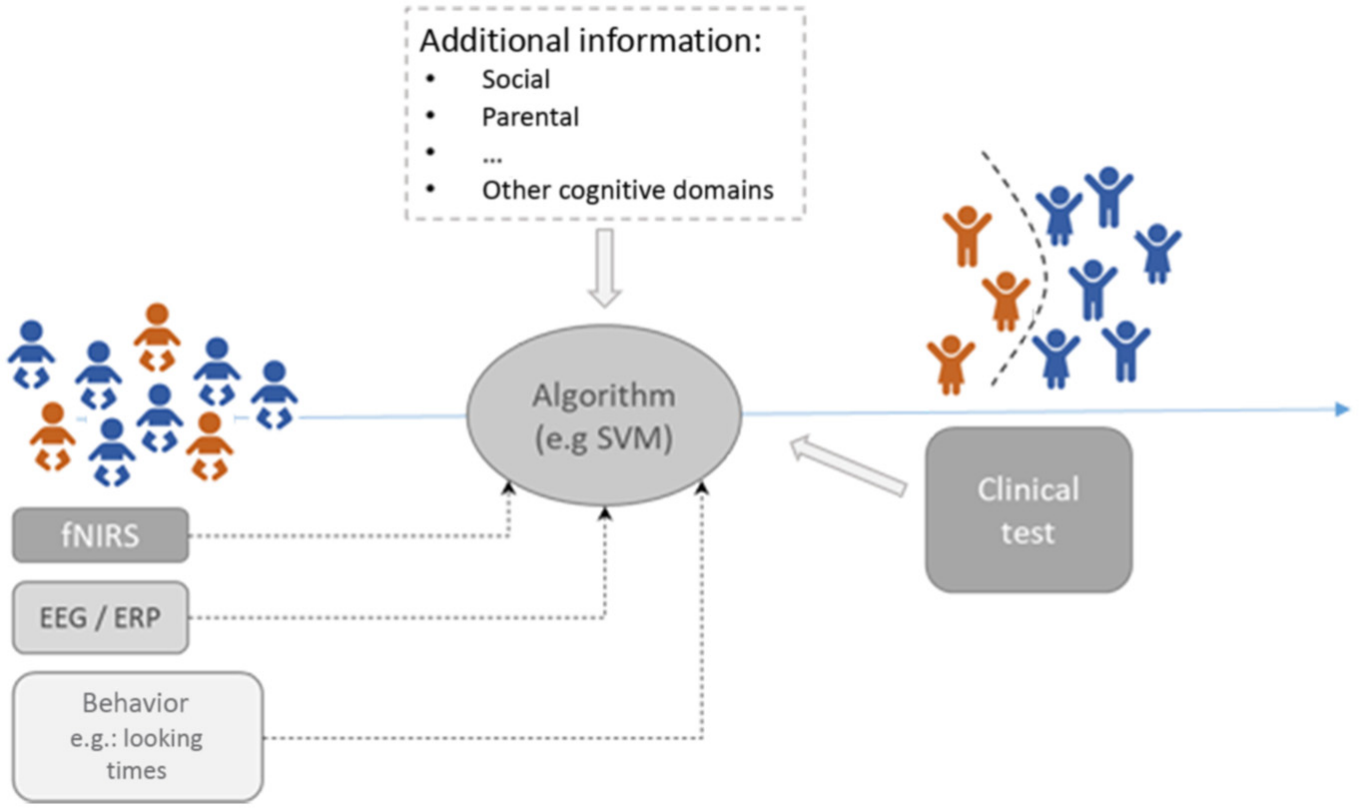
Potential design for supporting the prediction of the occurrence (e.g., dyslexia) or persistence (e.g., stuttering) of a neurodevelopmental disability, clinical diagnosis (e.g., ADHD), or a response to a pharmaceutical treatment. fNIRS measurements could be a component of a multimodal assessment strategy associated with (later) clinical-behavioral testing to diagnose the condition. Longitudinal assessment is mandatory for such an approach.

In terms of developmental disorders that affect language and communication, fNIRS may have the potential to improve prognostic evaluation. A “diagnostic” function, in the sense of providing a measurement to decide whether a disorder is present, appears to be currently unrealistic. It should be noted that this pertains to all functional imaging tools, as behavioral measurements in the form of standardized and psychometrically validated tests must be considered as the gold standard. (In other words: a child would not be considered dyslexic if his/her brain shows an abnormal activation pattern but rather if a test for reading/writing shows results outside the normative range). For the development of language and communication, even behavioral screening may be underused,^68^ especially for adolescents, making the more demanding assessment by functional imaging an unrealistic goal in a broader population. Therefore, a realistic target for future research is to use fNIRS in concert with other assessment tools to eventually sharpen the diagnostic criteria and prognosis for “at-risk” populations. For example, such predictive validity of ERPs has been shown for dyslexia.^69^^,^^70^ The diagram in Fig. 2 illustrates potentially fruitful designs that combine standardized assessment at an early age and correlating the results with later development.

In summary, the establishment of new clinically reliable tools for individual diagnosis/ prognosis is very rare. However, fNIRS may have the potential to contribute to group-wise predictions concerning the development or persistence of developmental deficits affecting language and communication. One great advantage of this methodology, especially in developmental language research, is the ease with which it can be combined with EEG and the fact that “natural” environments can be assessed. The option of simultaneously scanning multiple participants (hyper-scanning)^71^ may also be of interest to capture abnormalities in communication and social interaction. Aside from the conditions discussed in this section, this also applies to the following section on ADHD. This option has not yet been used in a clinical setting. Fascinating as it is, the challenge lies in the potential increase in variability between participants. To explore the clinical perspectives, however, large-scale studies ideally using similar paradigms for different languages and a correlation with later outcome appear to be the most promising avenues. Recent studies have documented the first steps in this direction for those who use CIs and the question of whether developmental stuttering will persist or resolve in later life.

## 4 Attention-Deficit/Hyperactivity Disorder (ADHD)

ADHD is one of the most common developmental disorders, affecting ∼5% of children.^72^ It is associated with a heavy societal and economic burden due to its high prevalence, its long-term functional consequences, and the frequent occurrence of psychiatric and developmental comorbidities that affect clinical outcomes. ADHD is characterized by persistent symptoms of inattention and/or hyperactivity-impulsivity that interfere with social, academic, or occupational functioning and/or development. Symptoms appear before the age of 12 years, are present in at least two settings (e.g., at home, school, or in social contexts), and cannot be explained by another condition, such as anxiety or intellectual disability.^73^

In the past 10 years, an increasing number of ADHD studies have used fNIRS. Similar to the use of fNIRS in developmental language research, the popularity of fNIRS in ADHD research stems from the comparatively low experimental constraints when applied to this population. Due to the intrinsic nature of the pathology, some children are unable to lie down and remain motionless in a cramped space, such as in an fMRI scanner. Furthermore, children with ADHD are at high risk of presenting with an additional neurodevelopmental disorder (e.g., ASD, dysphasia, anxiety, conduct disorder). Having the child seated comfortably in a chair or on his/her parent’s lap and allowing direct contact with the experimenter and the parents are major advantages in behaviorally and cognitively challenged pediatric populations for acquiring good-quality brain data.

### 4.1 What has been Studied?

The scoping review search yielded 151 entries. The search terms used in this section were: [(attention deficit disorder) OR (attention deficit) OR (ADD) OR (ADHD)]. All titles and abstracts were scanned and false and or redundant hits were removed. Fifty-two original articles remained and were reviewed in detail. For a summary of the main information related to these articles, please refer to Table S3 in the Supplementary Material. Among the 52 fNIRS papers on ADHD and children/adolescents, almost half (24/52) used fNIRS to better understand the pathophysiology of the disorder, only seven (7/52) used fNIRS for the screening or diagnosis of ADHD, and 21 used fNIRS to assess the impact or efficacy of an intervention, mostly pharmaceutical treatments. The main findings and global conclusions revealed by this body of literature are reported below.

#### 4.1.1 Pathophysiology of ADHD

Twenty-four fNIRS studies (20 in the last decade) have addressed the pathophysiology of ADHD. Among the 24 studies, 21 included fNIRS data recorded while participants were performing a cognitive task, most often a working memory task (e.g., n-back task) or an inhibitory control task (e.g., Go/no-Go task or Stroop task) and the analyses targeted the hemodynamic response to brain activation (HbO or HbO/HbR concentration changes). In these 21 articles, the head coverage was relatively limited and typically targeted the prefrontal/frontal cortices and/or temporal areas. Overall, the qualitative analyses of these studies revealed consistent hypoactivity in the right lateral prefrontal cortex for all tasks. This finding is consistent with those of previous fMRI studies on brain responses during attention and executive tasks in children with ADHD (e.g., Refs. 74 and 75). The three remaining publications (3/24) reported data acquired using a greater coverage of the head and applying functional connectivity analyses. First, Sutoko et al.^76^ included task-based dynamic functional connectivity analyses for 21 school-aged children with ADHD and 21 age-matched typically developing children who completed a Go/no-Go task during fNIRS recording (16 sources and 14 detectors). The authors identified four connectivity states during the temporal course of the task. Children with ADHD tended to show less occurrence of the dominant connectivity state than typically developing individuals. Conversely, the two connectivity states that were less frequent in controls were more prevalent in the ADHD group. These results suggest atypical dynamic network recruitment to accommodate task demands in children with ADHD, providing a new perspective to explain the neuropathophysiology of this disorder. The two other papers, published by the same team, investigated the integrity of functional brain networks at rest using 80 fNIRS channels (24 sources and 28 detectors). They used graph theoretical analyses^77^ and multiscale entropy^78^ to characterize six functional brain networks. Hu et al. found less variability in brain signals in several cerebral functional networks (e.g., the default mode, frontoparietal, attention, and visual networks) in 42 children with ADHD than in 41 healthy controls. This finding is consistent with a decrease in both functional connectivity and the efficiency of global networks reported previously by Wang et al. for 30 children with ADHD and 30 typically developing children using graph theory. Interestingly, the results from both studies correlated with ADHD scores or task performance indices, suggesting that such an approach could be useful to identify brain markers of symptomatology. Notably, the studies demonstrate the versatility and feasibility of using resting-state functional brain connectivity analyses in children with ADHD.

#### 4.1.2 Screening for or diagnosis of ADHD

The seven studies targeting screening or diagnosis of ADHD, all published in the past decade, included school-aged children and used machine learning algorithms.^76^^,^^79^^–^^84^ In 2020, Yasumura et al.^82^ published a multicenter study that included six healthcare facilities. They aimed to identify valid and objective markers of ADHD diagnosis using support vector machine algorithms. They included changes in HbO concentration during a reverse Stroop task and task performance in their age model on 170 children with ADHD and 145 typically developing controls and found an overall discrimination rate of 86.35%. As another interesting example, Sutoko et al.^76^ explored the efficacy of task-based cerebral activation features compared to task-based connectivity markers for ADHD screening using machine learning classification analyses in 36 children with ADHD and 23 typically developing children performing a visual oddball task. They showed the connectivity-based biomarker to perform better than the activation-based biomarker (88% versus 76% classification accuracy, respectively). Overall, these studies showed that the most discriminative brain areas for ADHD classification are the prefrontal, frontal, and temporal regions.^80^^,^^81^ These studies also documented relatively good diagnostic accuracy of over 80% (between 81% and 88%), suggesting that fNIRS could eventually contribute to improve diagnostic accuracy in ADHD based on machine learning approaches.

#### 4.1.3 Impact or efficacy of an intervention

Similar to studies on its potential clinical relevance, 21 studies used fNIRS to assess the impact or efficacy of an intervention. Most of these interventional studies (14/21) aimed to characterize the impact of a pharmaceutical treatment (methylphenidate or atomoxetine) on brain activity or cerebral organization using fNIRS and on ADHD symptomatology using questionnaires and behavioral measurements. Most studies reported an improvement or normalization of prefrontal hemodynamic activity associated with the treatment with a number of differences between medications^85^ and the genotype status of patients.^86^^,^^87^ However, many of the studies recorded brain responses from only the frontal or prefrontal areas, raising questions about the specificity of these results to the prefrontal areas.

Interestingly, four articles (4/21) aimed to identify fNIRS markers predictive of the clinical response to medication in children with ADHD. In 2021, Grazioli et al.^88^ used clustering analyses, including bilateral prefrontal and frontal HbO concentration changes, during an emotional continuous performance task, along with clinical and neuropsychological data, to characterize different types of responses to methylphenidate in 24 children with ADHD and 25 typically developing children. Their model identified distinct clusters (responses to medication), suggesting that such a model could eventually help to predict how a patient will respond to a pharmaceutical treatment. However, larger coverage (not only including the prefrontal and frontal areas) would again be more informative.

Finally, three publications targeted the utility of fNIRS-neurofeedback as a new method for treating ADHD. In a nonrandomized parallel-group study, Wu et al.^89^ compared the efficacy of fNIRS-neurofeedback to that of atomoxetine in children with ADHD. Eighteen children with ADHD aged between 8 and 12 years completed a 6-week fNIRS-neurofeedback intervention (12 sessions) and 31 age-matched children with ADHD received oral atomoxetine treatment. The results showed an improvement in behavioral parameters and clinical symptoms following fNIRS-neurofeedback and better efficacy of fNIRS-neurofeedback than atomoxetine treatment during and after the interventions. Although this study highlights a potential role and advantages of fNIRS-neurofeedback to treat ADHD, these results need to be replicated using a crossover, randomized protocol on a larger sample.

### 4.2 What are the Challenges?

There are several challenges and limitations associated with fNIRS research in ADHD. First, there is large heterogeneity between studies in terms of diagnostic criteria and the inclusion or exclusion of comorbidities. The diagnostic criteria for ADHD and its subtypes (i.e., inattentive, hyperactive/impulsive, combined) were most often not mentioned, and some studies included children with comorbidities (e.g., ASD), whereas others excluded them. This makes it difficult to compare the results between studies. In most publications, the sample sizes were relatively small, probably due to recruitment issues and the difficulty of acquiring good quality data from children with ADHD. These limitations preclude researchers from controlling for important sociodemographic and clinical variables and from drawing strong and generalizable conclusions. Although many studies made the effort to match groups for sex and age, most studies with nonmatching groups did not control for it in the analyses, leading to bias and even invalid interpretations in some cases. Furthermore, there have been no fNIRS studies on the effect of sex, age, or gender in children or adolescents with ADHD, which would be of interest in clinical care.

An important methodological limitation in most ADHD publications (but not limited to this clinical entity) is the highly limited scalp coverage. Most studies used probes placed only over the bilateral frontal areas, although some also included additional coverage of the temporal and/or parietal regions. Although the established impact of ADHD on executive functioning may justify focusing on the frontal/prefrontal areas, such narrow coverage precludes researchers from taking a large-scale network approach to study the spatial specificity underlying the pathophysiology of this disorder. This may be of particular relevance when targeting the effect of medication on brain hemodynamics for drugs that affect larger brain networks rather than only frontal brain regions, which was not brought to light in these articles. The underlying brain network of ADHD also probably involves deep cortical and subcortical structures that cannot be detected using fNIRS due to the shallow penetration of light, highlighting a general limitation of the technique in the investigation of pathological mechanisms, especially when they involve large-scale networks that support cognitive functioning.

### 4.3 What are Potential Perspectives?

Despite the above-mentioned limitations, we believe that the current fNIRS literature on ADHD offers several promising avenues for the development of approaches with a potential clinical impact. Using machine learning algorithms, fNIRS data combined with behavioral, clinical, and socio-demographic data offers relatively good diagnostic accuracy for ADHD (over 80%). fNIRS will surely not replace clinicians and behavioral testing for diagnosing ADHD, but it could eventually contribute by providing a computer-aided diagnostic tool to clinicians. This will be especially relevant in cases in which the clinical presentation is not typical or clear or in contributing to better defining the type of ADHD. Similar to its application in developmental disorders that affecting language and communication (see Sec. 3), the prediction of later development and the prognosis may be a prime target for fNIRS, ideally in concert with other neurophysiological and behavioral measurements (see Fig. 1). In terms of limitations, this literature is still young and the small cohort sizes included in most of the studies clearly call for an fNIRS database that includes typically and atypically developing children to build normative developmental fNIRS data. Another facet of the literature suggests that fNIRS may provide predictive markers for patients who will have a good response to medications, leading to a personalized medicine approach that could eventually be implemented in clinical practice. Not least, fNIRS-neurofeedback has been suggested as a promising nonpharmaceutical tool for the treatment of ADHD. However, only a nonrandomized parallel-group study and a pilot study have been published thus far. More evidence is still needed before considering offering fNIRS-neurofeedback in the clinical setting.

## 5 General Challenges and Perspectives of fNIRS in Pediatric Populations

The focused reviews of three conditions in infants, children, and young adolescents draw a mixed picture of the clinical use of fNIRS in pediatric populations. Although certain studies have exploited the strengths of the methodology, a number of studies have, at best, simply demonstrated the feasibility of certain approaches. Unfortunately, there is also a large number of studies in which the instrumentation, montage, and paradigms may have been inadequate for the question addressed. Harsh as this may sound, clinical applications ultimately require a procedure that aids diagnostic, prognostic, or interventional decisions. This implies that the result for an individual must be classified as either pathological or not. Standardized and operationalized statistical properties of a large reference group are generally required to reliably classify individual results. This has not been demonstrated by any of the reviewed studies. It should be noted, however, that the establishment of new methodologies for clinical use is extremely rare. Even for very broadly applied functional techniques, such as fMRI and ERP, approaches those aid clinical decisions are the exception. Therefore, the first step will be to target methodological and method-inherent limitations. These challenges and potential solutions, largely independent of the participants’ age, have been highlighted.^90^ Moreover, guidelines for best practices in fNIRS studies have been recently published^91^ and should be applied when designing any fNIRS study. Therefore, the community of fNIRS researchers can profit from a solid framework in which fruitful research may be expected in the near future. As clinical studies require large cohorts and normative data on neurotypical controls, a very promising step would be the establishment of repositories for data acquired in laboratories from around the world. Such an undertaking calls for simple and robust stimulation paradigms, ideally available to the world-wide fNIRS community. If the repository data are analyzed using modern classifier techniques, each case added will enhance the precision of classification. Such scenarios have been proposed for very common neurological diseases (e.g., Ref. 92), but might also serve the establishment of fNIRS for clinical use in the field of pediatrics.

We will not reiterate all the challenges and limitations of the previous sections. However, we would like to highlight a number of general challenges concerning the application of fNIRS. These include (i) the diversity of instrumentation used, (ii) the variability of coverage of brain areas, (iii) the variability of analysis strategies (including the issue of what combination of Hb parameters is used), (iv) the lack of a reference montage (e.g., 10 to 20 system provided for EEG), (v) the notorious issue of extra-cerebral signal contribution, (vi) the inherent limitation of fNIRS in spatial resolution (including the nonaccessibility of deep brain structures), and (vii) the still unsolved problem of hair (often restraining recordings to the frontal and prefrontal areas). Finally, an important challenge we have highlighted already is (viii) the choice of the stimulation used. Simplicity is highly advisable. This can be illustrated by an example from the field of language research, in which a paradigm addressing the detection and habituation to (non) adjacent dependencies in typically developing neonates was used.^93^ The study showed that newborns are sensitive to basic linguistic structure. Using an artificial grammar paradigm, the response to ABB syllable strings was larger than that to ABC strings, suggesting very early sensitivity to structure in linguistic input. Notably, in a study on high- versus low-ASD-risk children, the expected differential response to this stimulus was not found. However, this was partially due to the fact that the basic contrast (ABB- versus ABC syllable triplets) did not replicate in the control group.^94^ Although exploiting the full spectrum of (subtle) contrasts in neurodevelopmental research, in general, is of undoubted value, clinically oriented studies may be advised to use paradigms that have been used by several groups in typically developing children, with ideally rather large effect sizes. The very subtle difference shown in the original publication on neurotypical neonates^93^ may not be ideal to differentiate between clinical groups.

In summary, most of the challenges and sources of heterogeneity between studies are not restricted to the developmental and clinical literature. However, they constitute major obstacles to drawing conclusions and the development of tools that can then be translated and used in a clinical framework.

In addition, rather than allowing for individual or even subtype classification, fNIRS provides a better understanding of the temporal and spatial mechanisms involved in different pathologies. At the group level, the extraction or even identification of new hemodynamic neurobiomarkers for certain pathologies is possible. It must be kept in mind that methodologies such as fNIRS and fMRI are based on the activation of vascular networks. This response modality is secondary to neuronal activation and shows a delay of some 5 s. Thus, the neuronal processes that trigger the vascular response are considerably faster. However, the dynamics of the adaptation of the vascular system to neuronal activation may be specific to certain pathologies, in particular those of vascular origin. Therefore, alterations in neurovascular coupling may indeed be a promising target to better elucidate the mechanisms of certain pathologies.

## 6 Conclusions

Although the use of fNIRS in the clinical pediatric setting is currently very limited (mostly used as part of the presurgical workup of refractory epilepsy), the current literature offers several highly promising clinical avenues. The strong advantages of fNIRS relative to fMRI are its low constraints and portability. This makes it possible to provide information about the re/activity of the brain in a relatively natural environment. Such flexibility allows the design of new protocols that could be suitable for multimodal analysis that easily includes EEG, for example. Multimodal neuroimaging, such as fNIRS-EEG, and interdisciplinary assessments, including, for example, fNIRS, along with behavioral and clinical data, virtual reality, eye-tracking measurements, etc., embedded in a machine learning approach, could offer highly rich datasets to better understand a given neuropathophysiology or develop clinical tools for refining diagnoses and offering personalized intervention strategies. fNIRS hyperscanning is also developing rapidly and will certainly offer clinical possibilities for improving screening, diagnosis, and interventions in neurodevelopmental disorders or clinical conditions associated with social dysfunction (e.g., ASD, communication disorders, traumatic brain injury). For this approach, the benefit of multimodal imaging is also of potential interest, at least to evaluate the EEG background activity of the pediatric population during the fNIRS recording. Given the numerous advantages of using fNIRS in children and behaviorally and cognitively challenged individuals and rapid developments in computational neuroscience and fNIRS methodology, pediatric clinical research teams will certainly pursue their work developing clinical tools, which will eventually make their way to the clinic. Ideally, a shared data repository from groups worldwide may improve the problem of small cohort sizes, especially when well-defined and homogeneous clinical populations are targeted.
