# Supplementary material for: Functional near-infrared spectroscopy in pediatric clinical research: Different pathophysiologies and promising clinical applications
Source: Neurophotonics. 2023 Mar 3;10(2):023517. doi: 10.1117/1.NPh.10.2.023517 (PMC9982436; doi:10.1117/1.NPh.10.2.023517)
Supplement: Supplementary file 1 [file NPh_010_023517_SD001.pdf]

Supplementary Table S1. fNIRS articles on pediatric epilepsy

	Authors	Objective	NIRS technics	Other modality or measure	NIRS measures	Region of interest	Number of probes	Age range	*N*	Type of epilepsy
**Interictal spikes**										
Interictal spikes	Aarabi et al., 2017 [1]	Evaluate different methods for NIRS [2] analysis for the neurovascular coupling surrounding the IES	FD NIRS cortical	ECoG	HbO, HbR	Epileptic region	2 emitters, 2 detectors	N/A	8 rats	N/A
	Bourel et al., 2019	Evaluate the neurovascular coupling surrounding the IES in BECTS patients	FD NIRS	EEG	HbO, HbR	Frontal lobe	16 emitters, 4 detectors	N/A	6	BECTS
	Machado et al., 2011	Evaluate NIRS detection methods for NIRS analysis of the neurovascular coupling induced by IES	FD NIRS	EEG	HbO, HbR	Epileptic region	21 emitters, 8 detectors	10 y	1	Focal epilepsies
	Manoochehri et al., 2017	Evaluate changes in the extracellular space and in the hemodynamic surounding the IES	FD NIRS, FOS	HR EEG	HbO, HbR	Frontal lobe	4 emitters, 16 detectors	10–14 y	3	Frontal lobe epilepsy
	Osharina et al., 2010	Evaluate the neurovascular coupling surrounding the IES	FD NIRS cortical	ECoG	HbO, HbR	Epileptic region	2 emitters, 2 detectors	N/A	8 rats	N/A
	Peng et al., 2014	Evaluate the hemodynamic response to IES	FD NIRS	EEG	HbO, HbR	Epileptic region	64 emitters, 16 detectors	10–62 y	40	Neocortical epilepsy (n= 15); Mesial temporal lobe epilepsy (n=25)
	Peng et al., 2016	Use patient specific hemodynamic response function in IES	FD NIRS	EEG	HbO, HbR	Epileptic region	64 emitters, 16 detectors	14–27 y	9	Focal epilepsies

	Pouliot et al., 2012	Evaluate nonlinear hemodynamic response to IES	FD NIRS	EEG	HbO, HbR	Epileptic region	64 emitters, 16 detectors	N/A	3	Focal epilepsies
**Seizures**										
Neonatal	Silas et al., 2012	Evaluate the hemodynamic response to neonatal seizure	CW NIRS	aEEG PaCO2, SaO2	TOI	Epileptic region	N/A	Fullterm neonates	1	Anoxo ischemia
	Wallois et al., 2009	Evaluate the hemodynamic response to neonatal seizure	FD NIRS	EEG	HbO, HbR, TOI	Epileptic region	2 multidistance patchs: 2* (4 emitters 1 detector)	Fullterm neonates	1	Anoxo ischemia
Partial	Haginoya et al., 2002	Evaluate the hemodynamic response to partial seizure	CW NIRS	EEG	HbO, HbR, CBV	Frontal	1 emitter, 1 detector	0–16 y	3	Complex partial seizure
	N Guyen et al., 2013	Evaluate the hemodynamic response to partial seizure	FD NIRS	EEG	HbO, HbR, CBV	Frontal	64 emitters, 16 detectors	10–45 y	9	Focal epilepsies
	Gallagher et al., 2008a	Evaluate hemodynamic changes and langauge lateraliztaion in a boy with refractory epilepsy	FD NIRS	EEG, SPECT, fMRI, MEG	HbO, HbR, CBV	Bilateral temporal area	128 NIRS channels	10 y	1	Focal epilepsy
	Watanabe et al., 2000	Evaluate the hemodynamic response to partial seizure	FD NIRS	EEG SPECT	HbO, HbR	Temporal/ Parietal	8 to 24 channels	8–40 y	12	Focal epilepsies
	Watanabe et al., 2002	Evaluate the hemodynamic response to partial seizure	CW NIRS	EEG SPECT	HbO, HbR, CBV	Temporal/ Parietal	24 channels	9–40 y	26	Focal epilepsies
Spasms	Bourel et al., 2017	Evaluate the hemodynamic response surrounding the spasms	FD NIRS	EEG	HbO, HbR, CBV	Frontal	2 multidistance patchs: 2* (4 emitters 1 detector)	6 wk–8 m	6	West syndrome
	Haginoya et al., 2002	Evaluate the hemodynamic response to spasms	CW NIRS	EEG	HbO, HbR, CBV	Frontal	1 emitter, 1 detector	6 m	3	West syndrome

	Munakata et al., 2004	Evaluate the hemodynamic response to spasms	CW NIRS	EEG SPECT	HbO, HbR, CBV	Frontal	8 emitters, 10 detectors	3–7 m	3	West syndrome
Absence	Haginoya et al., 2002	Evaluate the hemodynamic response to absence	CW NIRS	EEG	HbO, HbR, CBV	Frontal	1 emitter, 1 detector	3–6 y	2	Absence epilepsy
	Noorhashemi et al., 2022	Evaluate the hemodynamic response to absence seizures and the relationship between Dc shift and changes in CBF	FD NIRS	EEG, DCS, FOS	HbO, HbR, CBV	Frontal	1 multidistance patchs (4 emitters 1 detector), 1 DCS emitter and 1 DCS detector, 1EEG	6–10 y	8	Absence epilepsy
	Roche Labarbe et al., 2008	Evaluate the hemodynamic response surronding the absence	FD NIRS	EEG	HbO, HbR, CBV	Frontal	2 multidistance patchs: 2* (4 emitters 1 detector)	1–16 y	6	Absence epilepsy
**Seizure detection procedures in children**	Sirpal et al., 2019	Study weither fNIRS improves seizure detection in multimodal EEG-fNIRS recordings	FD NIRS	EEG	HbO, HbR	Epileptic region	64 emitters, 16 detectors	11–62 y	40	Focal epilepsies
**Characterization of eloquent areas**	Gallagher et al., 2007	NIRS as an alternative to the IAT for language mapping	FD NIRS	fMRI, IAT	HbO, HbR	Temporal areas	64 emitters, 8 detectors	3–28 y	9	Normal and various epilepsy
	Gallagher et al., 2008a	Presurgical evaluation of language areas using fNIRS	FD NIRS	N/A	HbO, HbR	Whole head focusing temporal areas	16 emiters, 8 detectors	9 y	1	Focal epilepsies
	Gallagher et al., 2008b	Presurgical evaluation of language areas using fNIRS	FD NIRS	EEG	HbO, HbR	MEG SPECT MRI PET	128 channels (8 detectors, 16 multiplexed channel-source detector combinations)	10 y	1	N/A
	Gallagher et al., 2016	Language mapping in children using fNIRS resting state connectivity	FD NIRS	N/A	HbO, HbR	Whole head focusing temporal areas	64 emitters, 8 detectors	3–30 y	33	Focal epilepsies

Sato et al., 2012	Evaluate simultaneous NIRS and ECoG recordings during cortical stimulation	CW ETG 4100	ECoG	HbO, HbR, CBV	Temporal area	24 channels	18 y	1	Temporal lobe epliepsy
Sato et al., 2016	Evaluate the usefullness of cortico-cportical evoked hemodynamic response in human language system using intraoperative NIRS during direct cortical stimulation	CW ETG 7100	ECoG	HbO, HbR, CBV	Temporal area	24 channels	6 -28 y	3	Temporal lobe epliepsy
Vannasing et al., 2016	Study potential brain language reorganization in a boy with refractory epilepsy	FD NIRS	fMRI, IAT	HbO, HbR	Whole head focusing temporal areas	46 emitters, 11 detectors	6 and 10 y	1	N/A

[1] All articles included in the Supplementary Tables are reported in the reference list of the main text

[2] A list of all abreviations appearing in the Supplementary Tables is provided below

Abbreviations

aEEG: Amplitude electroencephalography

BECTS: Benign epilepsy with centrotemporal sikes

CBF: Cerebral blood flow

CBV: Cerebral blood volume

CW NIRS: Continuous wave near infrared spectroscopy

DCS: Diffuse correlation spectroscopy

ECoG: Electrocorticography

EEG: Electroencephalography

FD NIRS: Frequency domain near infrared spectroscopy

fMRI: Functional magnetic resonance imaging

FOS: Fast optical signal

HbO: Oxyhemoglobin

HbR: Deoxy-hemoglobin

HR EEG: High resolution electroencephalograph

IAT: Intracarotid amobarbital test

IES: Interictal epileptic spikes

m: Months

MEG: Magnetoencephalography

MRI: Magnetic resonance imaging

N/A: Not available (information not found in the article)

PaCO2: Partial pressure of carbon dioxide

PET: Positron emission tomography

SaO2: Oxygen saturation

SPECT: Single photon emission computed tomography

TOI: Tissue oxygenation index

wk: Weeks

y: Years

Supplementary Table S2. fNIRS articles on language and communicative impairments

Authors	Objective	NIRS Device	Other modality or measure	Task	NIRS measures	Regions of interest	Number of probes	Age range	*N*	Sex (M;F)
**Perception**										
Basura et al., 2018	Review on NIRS studies in CI-users and tinnitus	N/A	N/A	N/A	N/A	N/A	N/A	In CI: 3 studies in adults; 1 study in children	N/A	N/A
Bell et al., 2020a	Investigate what helps children to understand speech in noise and study the feasibility of a virtual auditory environment in combination with fNIRS	ETG-4000; Hitachi Medical Corporation	Behavioral measures	Comprehension of sentences in noise with different cues	HbT	Lager temporo-frontal area, target IFG/STG bilaterally	44 channels	avg 10 y	3 hearing impaired<br>6 controls	1;8
Bell et al., 2020b	Study neural basis of auditory and visual response-inhibition in children with hearing loss (and bilateral hearing aids) and children with ADHD	ETG-4000; Hitachi Medical Corporation	Behavioral measures	Visual and auditory go/no-go task	HbO, HbR	Bilateral frontal, temporal and parietal areas	44 channels	6–13 y	15 hearing-loss<br>20 ADHD<br>27 TDC	5;10 hearing-loss<br>14;6 ADHD<br>17;10 TDC
Bertachini et al., 2021	Study response to speech in congenital toxoplasmosis and control newborns	NIRScout Tandem; NIRx	Transiently evoked otoacoustic emissions and automated auditory brainstem responses	Four auditory-speech stimuli: mother infant direct speech, researcher infant directed speech, mother reading and researcher recorded	HbO, HbR	Full coverage	48 channels	18–94 days Congenial toxoplasmosis group<br>17–90 days TDC	38 Congenial toxoplasmosis<br>23 TDC	27;16 Congenial toxoplasmosis<br>12;11 TDC
Bortfeld H., 2019	Review of efforts to establish fNRIS in CI-users especially children	N/A	N/A	N/A	N/A	N/A	N/A	N/A	N/A	N/A
Calmels et al., 2022	Investigate auditory cortical activation in children with single-sided deafness and TDC	Protocol only	Assessment batteries for hearing in children	Binaural tasks	Lateralization of fNIRS response	Bilateral auditory cortex	18 channels	5–16 y	30 single-sided deafness<br>30 TDC	N/A

Dewey et al., 2015	Determine whether high-contrast moving visual stimuli and vibrotactile stimulation of the palms and fingers of both hands induced responses consistent with cross-modal plasticity in profoundly-deaf individuals	ETG-4000; Hitachi Medical Corporation	No	Auditory, visual and soatosensory stimuli	HbO, HbR	STG and neigh-bouring areas	24 channels	20–59 y deaf group<br>18–60 y TDC	30 deaf<br>30 TDC	12;18 deaf group<br>12;18 TDC
Harrison et al., 2021	Review of fNIRS in CI	N/A	N/A	N/A	N/A	N/A	N/A	7 articles in adults<br>1 article in children	N/A	N/A
Mushtaq et al., 2020	Identify an appropriate functional baseline for speech-specific processing using fNIRS in normal hearing school-age children	ETG-4000; Hitachi Medical Corporation	No	Press a button when a tone was heard during three auditory stimulus conditions (normal speech, time-reversed speech and signal-correlated noise	HbO, HbR	Bilateral temporal and frontal brain regions	44 channels	6–12 y	25	10;15
Saksida et al., 2022	Review of methods	N/A	N/A	N/A	N/A	N/A	N/A	N/A	N/A	N/A
Saliba et al., 2016	Review of methodology for CI and comparison to other techniques	N/A	N/A	N/A	N/A	N/A	N/A	Children and adults	N/A	N/A
Sevy et al., 2010	Ability of fNIRS to detect cortical responses to speech stimuli in pediatric CI users	NIRS 2CE; TechEn	fMRI	Segments of auditorily presented stories	HbO, HbR (comparison with BOLD)	Bilateral auditory cortices	4 channels	24–48 y TDC adults<br>4–15 y TDC children<br>2–19 y deaf children with at least 4 months experience with CI<br>2–8 y deaf children tested on the day of CI initial activation	11 TDC adults<br>12 TDC children<br>40 deaf children with at least 4 months experience with CI<br>13 deaf children tested on the day of CI initial activation	N/A
Wang et al., 2021	Assess emotional prosody processing pre-/post CI implantation	NirSmart, Huichuang,	Behavioral measures	Response to 4 emotions: fear, anger, happiness, and neutral (presented in pseudosentences)	HbO	Perisylvian with large coverage	52 channels	13–38 m	22	12;10

Wang et al., 2022	Differences in trajectories of activation patterns after right vs. left monaural CI	NIRSport2; NIRx	Behavioral measures	Listen to audio-book-segments vs. noise	HbO, HbR	Perisylvian bilateral	20 channels	avg 36.8 m hearing-loss group<br>avg 22.8 y TDC	34 hearing-loss group<br>35 TDC	23;11 hearing-loss group<br>15;20 TDC
**Comprehension**										
Anderson et al., 2017	Investigate if the variability in cerebral oxygenation patterns correlate with cognitive development in infants	CW NIRS; fNIRS Devices LLC	No	Watch a movie	StO2 and laterality index	PFC	16 channels	18–43 m	8 delayed<br>21 TDC	4;4 delayed<br>14;7 TDC
Edwards et al., 2017	Investigate potential endophenotypes of ASD risk present in the first 6 months of life	ETG-4000; Hitachi Medical Corporation	EEG	Differentiation and habituation to ABB vs ABC syllable strings	HbO	Bilateral perisylvian	24 channels	avg 3.58 m high risk of ASD<br>avg 3.62 m low risk of ASD	21 high risk of ASD<br>17 low risk of ASD	13;8 high risk of ASD<br>10;7 low risk of ASD
Kovelman et al., 2012	Understand how slow rhythmic sensitivity relates to language and reading acquisition	ETG-4000; Hitachi Medical Corporation	Behavioral measures	Word pair judgement: same/different and rhyme/no rhyme	HbO	Bilateral temporal, parietal and frontal areas	44 channels	6–9 y	15	11;4
Lawrence et al., 2021	Study typical fNIRS repsonse to speech, varied along intelligibility (vocoding)	ETG-4000; Hitachi Medical Corporation	Behavioral measures	Speech recordings<br>1. Noise<br>2. Noise-vocoded low 3. Noise vocoded high<br>(i.e.: 0, 50, 100% intellligibility)	HbO, HbR	Bilateral emporal and frontal cortex (perisylvian)	13 channels	6–13 y	19	6;13
Mao et al., 2021	Study consistency of evoked response for speech tokens and their discrimination by fNIRS response	NIRScout; NIRX	No	Speech tokens (consonant-vowel syllables) habituation/dis-habituation	HbO, HbR	Perisylvian	16 channels	2–10 m	23	16;7
Mahmoudzadeh et al., 2018	Investigating neuro vascular coupling in premature using speech sound differentiation stimuli	Imagent; ISS Inc.	EEG (31-61 electrodes)	Phonetic contrast (/b/ vs. /g/ and timbre (male/ female)	HbO, HbR, StO2	Bilateral perisylvian	20 channels	avg 30.5 wk	8 preterms<br>12 NIRS TDC<br>18 EEG TDC	6;2 preterms<br>8;4 NIRS TDC<br>11;7 EEG TDC
Marks et al., 2021	Study reading abilities depending on morphological awareness in children	CW6; TechEn	Reading abilities	Word-matching based on morphematic analysis	HbO	Bilateral frontal, post-temporal and parietal areas	46 channels	6–11 y	97 (24 reading impaired)	48;49

Minagawa-Kawai et al., 2009	Investigate ASD impact on lateralization of linguistic contrasts	ETG-7000; Hitachi Medical Corporation	No	Phonetic contrast (/itte/ vs. /itta/) and prosodic contrast (/itta/ vs. /itta?/)	HbT	Bilateral temporal areas, auditory cortex	8 channels	6–11 y ASD<br>5–9 y TDC	9 ASD<br>9 TDC	7;2 ASD<br>7;2 TDC
Mushtaq et al., 2019	Establish a baseline to fNIRS response to speech comprehnsion	ETG-4000; Hitachi Medical Corporation	No	Sentences / time-reversed speech / signal-correlated noise	HbO, HbR	Bilateral perisylvian	44 channels	6–12 y	25	10;15
Perdue et al., 2017	Study if heart rate response to speech stimuli is different between ASD low and high risk infants	ETG-4000; Hitachi Medical Corporation	Heart rate	Stimuli with nonsense 3 syllable strings	Heart rate extracted from HbO	No cerbral signal analysed	44 channels	3, 6, 9 and 12 m (longitudinal)	40 high-risk ASD<br>48 low-risk ASD	Variable across age groups
**Production**										
Hosseini et al., 2018	Define a classifier based on fNIRS features to differentiate children who stutter from those who don't	CW6; TechEn	Behavioral measures	Picture description (overt speaking)	HbO, HbR	IFG, STG, motor cortex	18 channels	7–11 y children who stutter and don't stutter<br>8–16 y children with recovered stutter	16 children who stutter<br>16 children who don't stutter<br>14 children with recovered stutter	13;3 children who stutter<br>11;5 children who don't stutter<br>10;4 children with recovered stutter
Jackson et al., 2021	Exploratory study in children with stutter	CW6; TechEn	Behavioral measures	Speech planning (non-word repetition) and speech execution (picture naming; overt/covert)	HbO, HbR	Motor cortices including SMA and reaching to bilateral temporal	46 channels	9–12 y	6 children with stutter<br>6 TDC	3;3 children with stutter<br>3;3 TDC
Jackson et al., 2019	Investigate activation patterns in people who stutter and compare it to PET/ fMRI findings	CW6; TechEn	PET, fMRI	Speech planning (non-word repetition) and speech execution (picture naming; overt/covert)	HbO, HbR	Central (motor/SMA) and bilateral perisylvian	36 channels (4 short channels)	avg 27 y adults with stutter<br>avg 26 y TDC	15 adults with stutter<br>15 TDC	11;4 adults with stutter<br>12;3 TDC
Kovelman et al., 2009	Investigate if bilingual production is dominated by executive control or also recruits language related areas, studied for bilingual and bimodal (spoken/signed) language production	ETG-4000; Hitachi Medical Corporation	Structural MRI for co-registration	Picture naming, for bilinguals: in mono- or bilingual mode (signing plus speech output)	HbO, HbR (PCA based)	Bilateral perisylvian/ coregistration with structural MRI	44 channels	16–32 y bilinguals ASL-English<br>19–42 y monolingual ASL<br>18–25 y monolingual English	5 bilinguals ASL-English<br>7 monolingual ASL<br>20 monolingual English	N/A

Kuwabara et al., 2006	Study cerebral activation differences in response to verbal fluency task between participants with/ without persistent developmental disorders	ETG-100; Hitachi Medical Corporation	Behavioral measures	Verbal fluency task vs. simple repitition of letters	HbO, HbR	Bilateral frontal and prefrontal cortices	24 channels	18–37 y persistent developmental disorder<br>24–34 y TDC	10 persistent developmental disorder<br>10 TDC	6;4 persistent developmental disorder<br>9;1 TDC
Mercure et al., 2020	Study differences in language processing depending on whether input was monolingual (spoken), bilingual-unimodal (spoken/spoken), or bilingual-bimodal (spoken/signed)	NTS OT-system; Gowerlabs	Looking times (to exclude trials)	Audiovisual clips with 4 conditions: 1. spoken-English; 2. spoken-French; 3. British sign language; 4. French sign language. 1. and 2. are (partially) known; 3. and 4. are foreign to all participants	HbO, HbR (unidirectional changes in both parameters excluded)	Bilateral perisylvian	46 channels	4–8 m	19 monolingual-spoken<br>20 bilingual-spoken<br>21 bilingual-bimodal (spoken/ASL)	11;8 monolingual-spoken<br>14;6 bilingual-spoken<br>10;11 bilingual-bimodal (spoken/ASL)
Sato et al., 2011	To find out about altered lateralization for linguistic contrasts in people who stutter	ETG-100; Hitachi Medical Corporation and OMM-2001; Shimadzu	Behavioral measures	Phonetic contrast (/itte/ vs. /itta) and prosodic contrast (/itta/ vs. /itta?/)	HbT	Bilateral perisylvian	24 channels	18–44 y adults<br>6–12 y school aged children<br>3–5 y preschool aged children	10 adults<br>7 school aged children<br>6 preschool aged children	10;0 adults<br>5;2 school aged children<br>5;1 preschool aged children
Tichenor et al., 2022	Study the consistency of the hemodynamic recponse over speech and language brain regions	TechEn device	Behavioral measures	Picture description task	HbO, HbR	Bilateral perisylvian	18 channels	7–12 y	37	20;17
Walsh et al., 2017	Investigate cortical activation differences between children who stutter and those who don't	TechEn device	Behavioral measures	Picture description (overt)	HbO, HbR	IFG, STG, (pre)motor	18 channels	7–11 y	16 children who stutter<br>16 TDC	13;3 children who stutter<br>11;5 TDC
**Reading/writing**										
Gallagher et al., 2008	Determine if language lateralization be assessed by fNIRS in presurgical evaluation of intractable epilepsy in children	Imagent; ISS Inc.	Behavioral measures	Verbal fluency and listening to stories presented in blocks	HbO, HbR	Bilateral perisylvian	128 channels	9 y	1	1;0

Gallagher et al., 2007	Determine if fNIRS show similar results when compared to IAT test and fMRI regarding the lateralization of language processing	Imagent; ISS Inc.	fMRI in selected participants<br>IAT in some participants	Verbal fluency versus nonsense-syllable repetition (latter is high level baseline)	HbO, HbR	Left temporal and frontal language areas and right hemispheric homologues	76-98 channels (64 emitters, 8 detectors)	3-15 y children<br>18-29 y adults	6 children<br>5 adults	8;3
Pecyna and Pokorski, 2013	Examine the oxygenation over prefrontal cortex as an indicator of dyslexia	FlexComp Infiniti/ BioGraph Infinity V4.0 ; Thought Technology Ltd	No	No task but training (10 d 20 mins /d) between measurements, measurements pre-/post-training resting state	Blood-oxygenation (NIR-HEG opto-technique, multiple parameters)	N/A	N/A	6–7 y group 1<br>9–10 y group 2<br>19–20 y group 3	16 group 1<br>16 group 2<br>16 group 3	8;8 group 1<br>8;8 group 2<br>8;8 group 3
Sela et al., 2014	Explore the lexical decision task in children/ young adults/ young dyslectic adults; study how does activation differ over left PFC	fNIRS Devices LLC	Behavioral measures	Lexical decision (words/ pseudowords) in a reading task	HbO	PFC (underlying forehead)	16 channels (only 1 analysed)	avg 12.7 y TDC<br>avg 25.7 y dyslexic adults<br>avg 25.1 y typical adults	17 TDC<br>17 dyslexic adults<br>17 typical adults	9;8 TDC<br>9;8 dyslexic adults<br>8;9 typical adults
Song et al., 2012	Investigate changes in cortical response to phonological tasks in dyslexic children	Custom made: Huazhong University of Science and Technology, Wuhan, China	Behavioral measures	Reading: identity judgement of written word: consonant/vowel; exchange of consonant/ vowel in 2 written words	HbO, HbR, HbT	Prefrontal areas covered by strip-arrangement	16 channels	avg 10.1 y dyslexics<br>avg 10.4 y controls	20 dyslexics<br>20 controls	11;9 dyslexics<br>13;7 controls
Wang et al., 2020	Review cognitive studies with NIRS in preterm infants (speech perception; motor performance; facial recognition)	N/A	N/A	N/A	N/A	N/A	N/A	N/A	5 studies on speech perception included in review of 13 stuies	
Zhu et al., 2012	Not clearly stated, but most likely correlation between working memory and dyslexia	Custom made 16 channels strip	Behavioral measures	Calculation (addition) and recall of numbers presented visually	HbT	Left PFC (selectively measured)	16 channels	avg 10.3 y dyslexics<br>avg 10.5 y controls	12 dyslexics<br>12 controls	N/A

Abbreviations

ASL: American sign language

ADHD: Attention-deficit/hyperactivity disorder

ASD: Autism spectrum disorder

avg: Average

CI: Cochlear implant

CW NIRS: Continuous wave near infrared spectroscopy

EEG: Electroencephalography

fMRI: Functional magnetic resonance imaging

HbO: Oxyhemoglobin

HbR: Deoxy-hemoglobin

HbT: Total hemoglobin

IAT: Intracarotid amobarbital test

IFG: Inferior frontal gyrus

m: Months

MRI: Magnetic resonance imaging

N/A: Not available (information not found in the article)

PET: Positron emission tomography

PFC: Prefrontal cortex

SMA: Supplementary motor area.

STG: Superior temporal gyrus

StO2: Tissue oxygen saturation

TDC: Typically developing controls

wk: Weeks

y: Years

Supplementary Table S3. fNIRS articles on ADHD

Authors	Objective	NIRS Device	Other modality or measure	Task	NIRS measures	Regions of interest	Number of probes	Age range	*N*	Sex (M;F)
**Pathophysiology**										
Arai et al., 2016	Study developmental changes in the neural underpinnings of self-generated spatial working memory in children with ADHD	OEG-16 head module; Spectratec Inc.	Behavioral measures	Spatial working memory task	HbO	Bilateral prefrontal areas	16 channels (6 emitters, 6 detectors)	7-13 y	30 ADHD<br>35 TDC	30;0 ADHD<br>35;0 TDC
Calub et al., 2022	Compare attention control abilities in children with ADHD and TDC	NIRSport, NIRx Medical Technology LLC	Behavioral measures	Visual array task	HbO	Bilateral PFC	20 channels (8 emitters, 8 detectors)	8-12 h	19 ADHD<br>18 TDC	16;3 ADHD<br>N/A TDC
Grazioli et al., 2019	Investigate the association between polyunsaturated fatty acids biostatus and cerebral cortex metabolism measured by fNIRS in a sample of children with and without ADHD	DYNOT Compact 9-32; NIRx	Poly-unsaturated fatty acids biostatus, behavioral measures	N-back working memory task	HbO, HbR	Bilateral fronto-temporal areas	32 channels (8 emitters, 24 detectors)	8-14 y	24 ADHD<br>22 TDC	24;0 ADHD<br>20;1 TDC
Gu et al., 2017	Investigate the relationship between the permutation entropy values and the cortical activations, and the different permutation entropy values between the children with and without ADHD	ETG-4000; Hitachi Medical Corporation	Behavioral measures	N-back working memory task	HbO	Bilateral VLPFC, DLPFC, presylvian frontal cortex, temporal cortex	52 channels (17 emitters, 16 detectors)	6-9 y	15 ADHD<br>16 TDC	10;5 ADHD<br>10;6 TDC
Hu et al., 2021	Explore the utility of multiscale entropy to measure brain signal variability in fNIRS	Multichannel near-infrared optical imaging system (Hui Chuang, China)	Behavioral measures	None	HbO	Whole brain (results in frontal, parietal and occipital cortices)	80 channels (24 emitters, 28 detectors)	8-12 y	42 ADHD<br>41 TDC	42;0 ADHD<br>41;0 TDC
Ichikawa et al., 2014	Examine the distinctive cerebral hemodynamics of ADHD and TDC children while they viewed happy and angry expressions	ETG-4000; Hitachi Medical Corporation	No	Emotional facial expression recognition task	HbO	Superior temporal sulcus	24 channels (5 emitters, 4 detectors)	8-12 y	13 ADHD<br>13 TDC	13;0 ADHD<br>13;0 TDC

Inoue et al., 2012	Examine the hemodynamic response during the go/no-go task in children with and without ADHD	Cognoscope; NIM Inc.	Behavioral measures	Go/no-go task	HbO, HbR	Bilateral frontal areas	16 channels (4 emitters, 10 detectors)	6-14 y	20 ADHD<br>20 TDC	14;6 ADHD<br>14;6 TDC
Ishii et al., 2017	Investigate frontal inhibitory function in children with ADHD	ETG-4000; Hitachi Medical Corporation	Behavioral measures	Rock, paper, scisors task	HbO	Frontopolar and dorsolateral cortex	22 channels (8 emitters, 7 detectors)	6-16 y	18 ADHD<br>27 TDC	17;1 ADHD<br>13;14 TDC
Kaga et al., 2020	Investigate the neurophysiological biomarkers for deficits in executive function in children with ADHD using fNIRS and EEG	OEG-16 head module; Spectratec Inc.	EEG and behavioral mesures	Colored go/no-go task	HbO	Bilateral PFC	16 channels (6 emitters, 6 detectors)	8-11 y	20 ADHD<br>18 TDC	17;3 ADHD<br>11;7 TDC
Mauri et al., 2020	Evaluate sensitivity to emotional stimuli in children with ADHD using fNIRS	DYNOT Compact 9-32; NIRx	Behavioral measures	Visual continuous performance task with stimuli of different emotional content	HbO, HbR	Bilateral prefrontal and frontal areas	6 emitters, 11 detectors	6-16 y	18 ADHD<br>25 TDC	2;16 ADHD<br>4;21 TDC
Miao et al., 2017	Demonstrate whether fNIRS can detect the changes in the concentration of HbO in children with ADHD and TDC	ETG-4000; Hitachi Medical Corporation	Behavioral measures	Go/no-go task	HbO	Bilateral PFC	52 channels (17 emitters, 16 detectors)	6-9 y	14 ADHD<br>15 TDC	10;4 ADHD<br>11;4 TDC
Moser et al., 2009	Explore if brain activation is altered during an event-related color-word matching Stroop task in comparison to a healthy control group	NIRO-300 spectrometer; Hamamatsu Photonics	Behavioral measures	Color-word matching Stroop task	HbO, HbR	Lateral PFC	2 channels	8-13 y	12 ADHD<br>12 TDC	12;0 ADHD<br>12;0 TDC
Negoro et al., 2010	Assess prefrontal brain dysfunction in ADHD children	ETG-4000; Hitachi Medical Corporation	Behavioral measures	Stroop color-word task	HbO	Inferior lateral PFC bilaterally	24 channels	6-13 y	20 ADHD<br>20 TDC	18;2 ADHD<br>17;3 TDC
Sanefuji et al., 2014	Understand the neural basis of ADHD by investigating short-term memory strategies using NIRS	OMM-2001; Shimadzu	Behavioral measures	Short-term memory for visual or phonological objects task	HbO, HbR, HbT	Left VLPFC	4 channels	6-12y	10 ADHD<br>19 TDC	9;1 ADHD<br>9;10 TDC

Schecklmann et al., 2010	Study if object and spatial visual working memory are distinctly impaired in ADHD	ETG-4000; Hitachi Medical Corporation	Behavioral measures	Visual working memory tasks	HbO	Bilateral prefrontal areas	52 channels (17 emitters, 16 detectors)	8-15 y	19 ADHD<br>19 TDC	17;2 ADHD<br>15;4 TDC
Shimamura et al., 2019	Investigate the recognition of familiar and unfamiliar faces in children with ADHD	ETG-4000; Hitachi Medical Corporation	Behavioral measures	Familiar and unfamiliar face recognition task	HbO, HbR, HbT	Bilateral temporal regions	24 channels	7-13 y	9 ADHD<br>14 TDC	9;0 ADHD<br>14;0 TDC
Sutoko et al., 2020	Study shifted brain connectivity under externally evoked stimulus using NIRS in children with ADHD	ETG-4000; Hitachi Medical Corporation	Behavioral measures	Go/no-go task	HbO, HbR	Prefrontal to inferior parietal lobes	16 emitters, 14 detectors	avg 7.8 y ADHD<br>avg 8.5 y TDC	21 ADHD<br>21 TDC	17;4 ADHD<br>15;6 TDC
Suzuki et al., 2017	Examine activity in the superior frontal cortex during the flanker task in children with ADHD and children with typical development using NIRS	OEG-16 head module; Spectratec Inc.	Behavioral measures	Flanker task	HbO	Bilateral superior frontal cortex	16 channels (6 emitters, 6 detectors)	8-11 y	12 ADHD<br>14 TDC	11;1 ADHD<br>10;4 TDC
Tsujimoto et al., 2013	Investigate the effect of distraction on working memory and its underlying neural mechanisms in children with ADHD	OEG-16 head module; Spectratec Inc.	Behavioral measures	Working memory task (with and without distraction)	HbO	Lateral PFC	16 channels (6 emitters, 6 detectors)	10.9 y ADHD<br>10.1 y TDC	16 ADHD<br>10 TDC	16;0 ADHD<br>10;0 TDC
Wang et al., 2020	Assess the value of resting-state fNIRS as an imaging technique to record spontaneous brain activity in children with ADHD	Multichannel continuous wave near-infrared optical imaging system (Hui Chuang, China)	Behavioral measures	None	HbO	Whole brain connectivity and networks, primary regions of the whole head (frontal, temporal, parietal, and visual cortexes)	24 emitters, 28 detectors	7-12 y ADHD	30 ADHD<br>30 TDC	30;0 ADHD<br>30;0 TDC
Weber et al., 2005	Evaluate the hemodynamic changes in both prefrontal regions induced by a cognitive task in children with ADHD in comparison to normal controls using NIRS	NIRO-300 spectrometer; Hamamatsu Photonics	Behavioral measures	Trail making task	HbO, HbR, TOI, CBV	Bilateral PFC	2 optodes	avg 10.4 y ADHD<br>avg 11.3 y TDC	11 ADHD<br>9 TDC	11;0 ADHD<br>9;0 TDC

Xiao et al., 2012	Compare inhibitory control between children with high functioning autism and those with ADHD	JH-NIRS-BR-05; Huazhong University of Science and Technology	Behavioral measures	Go/no-go and Stroop tasks	HbO	Bilateral PFC	16 channels	8–14 y	19 ASD<br>16 ADHD<br>16 TDC	19;0 ASD<br>16;0 ADHD<br>N/A for TDC
Yasumura et al., 2014	Evaluate the characteristics of children with ADHD using the Stroop task and reverse Stroop task that reflects the inhibition function of executive function	OEG-16 head module; Spectratec Inc.	Behavioral measures	Computer-based Stroop test and reverse Stroop test	HbO	PFC	16 channels (6 emitters, 6 detectors)	avg 11.18 y ADHD	10 ADHD<br>11 ASD<br>15 TDC	8;2 ADHD<br>7;4 ASD<br>6;9 TDC
Yasumura et al., 2019	Explore changes in PFC function in children with ADHD	OEG-16 head module; Spectratec Inc.	Behavioral measures	Reverse Stroop task	HbO	Bilateral PFC	16 channels (6 emitters, 6 detectors)	6-12 y	67 ADHD<br>140 TDC	58;9 ADHD<br>65;75 TDC
**Screening or Diagnosis**										
Gu et al., 2018	Use fNIRS activation patterns to identify ADHD children from TDC	ETG-4000; Hitachi Medical Corporation	Behavioral measures	N-back task	HbO	mPFC, DLPFC, VLPFC, posterior superior frontal cortex, superior temporal cortex	52 channels (17 emitters, 16 detectors)	6-9 y	25 ADHD<br>25 TDC	16;9 ADHD<br>16;9 TDC
Ichikawa et al., 2014	Classify the hemodynamic data and predict to which diagnostic group an unknown participant belongs for ADHD and ASD	ETG-4000; Hitachi Medical Corporation	No	Watch image of the child's mother's face and an unknown female face	HbO	Bilateral temporal area centered at T5 and T6 according to the International 10–20 system	24 channels	avg 9y9m ADHD<br>avg 9y9m ASD	9 ADHD<br>8 ASD	9;0 ADHD<br>8;0 ASD
Monden et al., 2015	Explore a method of individual differentiation between ADHD and TDC children using multichannel fNIRS, emphasizing how spatial distribution and amplitude of hemodynamic response are associated with inhibition-related right prefrontal dysfunction	ETG-4000; Hitachi Medical Corporation	Behavioral measures	Go/no-go task	HbO	Lateral PFC and inferior parietal lobe	22 channels (8 emitters, 7 detectors)	6-15 y	30 ADHD<br>30 TDC	25;5 ADHD<br>20;10 TDC

Sutoko et al., 2019a	Propose a classification method of differentially recognizing the ASD-comorbid condition in ADHD children	ETG-4000; Hitachi Medical Corporation	Behavioral measures	Go/no-go task	HbO	DLPFC and VLPFC	22 channels (8 emitters, 7 detectors)	avg 7.8 y ADHD avg 8.2 ASD-comorbid ADHD	21 ADHD 11 ASD-comorbid ADHD	17;4 ADHD 11;0 ASD-comorbid ADHD
Sutoko et al., 2019b	Exploring attentive task-based connectivity, compared to activation features, for screening ADHD children	ETG-4000; Hitachi Medical Corporation	Behavioral measures	Visual oddball task	HbO, HbR	Bilateral prefrontal to parietal cortices	44 channels (8 emitters, 7 detectors)	avg 9.5 y ADHD prescribed MPH avg 9.9 y ADHD prescribed ATX avg 9.8 y TDC	36 ADHD and ASD-ADHD 23 TDC	31;5 ADHD and ASD-ADHD 15;8 TDC
Yasumura et al., 2020	Establish valid, objective biomarkers for ADHD using machine learning	OEG-16 head module; Spectratec Inc.	Behavioral measures	Reversed Stroop task	HbO	Bilateral PFC	16 channels (6 emitters, 6 detectors)	avg 10.6 y ADHD avg 9.7 TDC	108 ADHD 108 TDC	92;16 ADHD 56;52 TDC
**Intervention**										
Araki et al., 2015	Examine the effects of long-term treatment with ATX on prefrontal hemodynamic activity in ADHD children during a continuous performance task using NIRS	ETG-4000; Hitachi Medical Corporation	Behavioral measures	Continuous performance test	HbO	Bilateral PFC including superior, middle and inferior frontal gyrus	24 channels	6-13 y ADHD 6–15 y TDC	12 ADHD 14 TDC	6;6 ADHD 5;9 TDC
Blume et al., 2017	Study protocol for a randomized controlled trial investigating the efficacy of neurofeedbacktraining NIRS for children with ADHD in a VR compared to a 2D environment	ETG-4000; Hitachi Medical Corporation	EMG, accelometer, virtual reality, behavioral measures	Immersive classroom environement	HbO	Bilateral DLPFC, prefrontal, central, temporal, and parietal areas of each hemisphere	24 optodes (14 emitters, 14 detectors)	6-10 y	90 (30 NIRS in VR, 30 NIRS in 2D, 30 EMG in VR)	N/A
Grazioli et al., 2021	Determine whether clustering analysis could be useful to characterize different clusters of responses to MPH in children with ADHD	N/A	Behavioral and clinical measures	Emotional continuous performance task	HbO	Bilateral prefrontal and frontal areas	14 channels	6-16 y	24 ADHD 25 TDC	N/A
Ishii-Takahashi et al., 2015	Develop a supplementary neuroimaging biomarker for predicting the clinical effect of continuous MPH administration by using NIRS	ETG-4000; Hitachi Medical Corporation	Behavioral measures	Stop signal task	HbO	Bilateral inferior frontal cortex	24 channels	8.6 y	30 ADHD 20 TDC	26;4 ADHD 14;6 TDC

Jang et al., 2021	Explore the acute effects of MPH on behavioral performance and brain activity during a VR-based working memory task simulating real-life classroom settings in ADHD children	NIRSIT; OBELAB	Virtual reality, behavioral measures	Working memory task (n-back task) in virtual class-room environement	HbO	Bilateral DLPFC, VLPFC, mPFC and orbitofrontal cortex	48 channels (24 emitters, 32 detectors)	7-16 y ADHD<br>7-14 y TDC	23 ADHD<br>12 TDC	16;7 ADHD<br>6;6 TDC
Kawai et al., 2021	Investigate the usefulness of NIRS for evaluating drug effects and improvements in medication adherence in children with ADHD	NIRStation OMM-3000-12; Shimazu	Behavioral measures	Stroop color-word test	HbO	Left and right forehead	2 channels	avg 9 y	10 ADHD<br>10 TDC	10;0 ADHD<br>10;0 TDC
Kobayashi et al., 2020	Investigate the possibility that acute administration of MPH affects processing of facial expressions in ADHD children	ETG-4000; Hitachi Medical Corporation	Behavioral measures	Facial emotion recognition task	HbO	Bilateral temporal areas	22 channels (8 emitters, 7 detectors)	8-12 y	19 ADHD	18;1
Li et al., 2022	Use fNIRS to preliminarily investigate the interaction of MPH treatment-related prefrontal inhibitory functional changes with the genotype status of the synaptosomal associated protein 25 gene in children with ADHD	NIRSport; NIRx Medical Technology LLC	Molecular/genetic analysis, behavioral measures	Go/no-go task	HbO, HbR	Bilateral PFC	16 channels (4 emitters, 12 detectors)	6-12 y	38 ADHD (27 T/T genotype, 11 G allele carriers [T/G + G/G])	29;9
Marx et al., 2015	Investigate NIRS-neurofeedback as a new method for the treatment of ADHD	ETG-4000; Hitachi Medical Corporation	EEG-neurofeedback, EMG-feedback, behavioral measures	NIRS neurofeedback	HbO	Frontal and temporal areas	44 channels	7-10 y	27 (9 NIRS, 9 EEG, 9 EMG)	18;9
Matsuura et al., 2014	Investigate the acute effects of MPH on neuropsychological performance and hemodynamic activation in children with ADHD during visuospatial working memory tasks	OEG-16 head module; Spectratec Inc.	Behavioral measures	Visuospatial working memory task	HbO	Bilateral PFC	16 channels (6 emitters, 6 detectors)	10.8 y	11 ADHD	10;1

Monden et al., 2012	Explore the feasibility of using fNIRS to search for a clinically implementable biological marker for the acute MPH effect on ADHD children	ETG-4000; Hitachi Medical Corporation	Behavioral measures	Go/no-go task	HbO	Lateral PFC	22 channels (8 emitters, 7 detectors)	7-14 y	12 ADHD	11;1
Nagashima et al., 2014a	Explore the neural substrate for MPH effects on attentional control in school-aged children with ADHD using fNIRS	ETG-4000; Hitachi Medical Corporation	Behavioral measures	Oddball task	HbO	Lateral PFC and inferior partietal lobes	22 channels (16 emitters, 14 detectors)	6-14 y	22 ADHD<br>22 TDC	19;3 ADHD<br>15;7 TDC
Nagashima et al., 2014b	Explore the neural substrate for ATX effects on attentional control in school-aged children with ADHD using fNIRS	ETG-4000; Hitachi Medical Corporation	Behavioral measures	Oddball task	HbO	Lateral PFC and inferior partietal lobes	22 channels (16 emitters, 14 detectors)	6-14 y	15 ADHD<br>15 TDC	12;3 ADHD<br>12;3 TDC
Nagashima et al., 2014c	Explore the neural substrate for effects of ATX on inhibitory control in school-aged children with ADHD using fNIRS	ETG-4000; Hitachi Medical Corporation	Behavioral measures	Go/no-go task	HbO	Lateral PFC and inferior parietal lobe	22 channels (8 emitters, 7 detectors)	6-14 y	16 ADHD<br>16 TDC	14;2 ADHD<br>14;2 TDC
Nakanishi et al., 2017	Examine differences in prefrontal hemodynamic activity between MPH and ATX in children with ADHD	ETG-4000; Hitachi Medical Corporation	Behavioral measures	Stroop color-word task	HbO	Frontal lobes	24 channels	6-14 y	30 ADHD	25;5
Öner et al., 2010	Investigate the interaction of treatment-related hemodynamic changes with genotype status for synaptosomal associated protein 25 gene in participants with ADHD on and off single dose short-acting MPH treatment with fNIRS	NIROXCOPE 301; Biophotonics Laboratory (Bogazici University)	Molecular analyses, behavioral measures	Color-word matching Stroop task	HbO, HbR, HbT	Bilateral DLPFC	16 channels (4 emitters, 10 detectors)	7-14 y (children)<br>18-43 y (adults)	31 ADHD (15 adults, 16 children)	8;7 adults<br>13;3 children
Ota et al., 2014	Examine the effects of a clinical dose of ATX on changes in prefrontal hemodynamic activity in children with ADHD, as measured by NIRS using the Stroop color-word task	ETG-4000; Hitachi Medical Corporation	Behavioral measures	Stroop color-word task	HbO	PFC	24 channels	7-13 y	10 ADHD	7;3

Schecklmann et al., 2011	Investigate olfactory sensitivity, discrimination, and identification in children with ADHD under chronic MPH medication and after a wash-out period	ETG-4000; Hitachi Medical Corporation	Behavioral measures	Olfactory task	HbO	Bilateral inferior frontal temporal brain areas	48 channels (16 emitters, 16 detectors)	10-16 y	27 ADHD 22 TDC	20;7 ADHD 8;14 TDC
Weber et al., 2007	Evaluate the feasibility of measuring cerebral hemodynamic effects of a clinical dose of MPH by NIRS in boys with ADHD	NIRO-300 spectrometer; Hamamatsu Photonics	Behavioral measures	Trail making task	HbO, HbR, HbT, TOI	DLPFC	2 channels	8-11 y	10 AHDH	10;0
Wigal et al., 2012	Determine reorganization of brain neurovascular properties following the medication treatment	Oximeter; ISS Inc	Behavioral measures	None	HbO, HbR	Left and right frontal areas	2 detectors, 10 emitters	6-12 y	26 ADHD	20;6
Wu et al., 2022	Assess the efficacy of NIRS real-time neurofeedback versus ATX in children with ADHD	LABNIRS system; Shimadzu Corp.	Behavioral measures	NIRS neurofeedback	HbO	Bilateral DLPFC	44 channels	8-12 y	49 ADHD (18 NIRS, 31 ATX groups)	13;5 NIRS 22;9 ATX

Abbreviations

ADHD: Attention-deficit/hyperactivity disorder

ASD: Autism spectrum disorder

ATX: Atomoxetine

avg: Average

CBV: Cerebral blood volume

DLPFC: Dorsolateral prefrontal cortex

EEG: Electroencephalography

EMG: Electromyography

h: Hours

HbO: Oxyhemoglobin

HbR: Deoxy-hemoglobin

HbT: Total hemoglobin

m: Months

mPFC: Medial prefrontal cortex

MPH: Methylphenidate

N/A: Not available (information not found in the article)

PFC: Prefrontal cortex

TDC: Typically developing controls

TOI: Tissue oxygenation index

VLPFC: Ventrolateral prefrontal cortex

VR: Virtual reality

y: Years

2D: Two-dimensional
